# Phytochrome regulates cellular response plasticity and the basic molecular machinery of leaf development

**DOI:** 10.1093/plphys/kiab112

**Published:** 2021-03-09

**Authors:** Andrés Romanowski, James J Furniss, Ejaz Hussain, Karen J Halliday

**Affiliations:** 1 Halliday Lab, Institute of Molecular Plant Sciences (IMPS), King’s Buildings, University of Edinburgh, Edinburgh, UK; 2 Comparative Genomics of Plant Development, Fundación Instituto Leloir (FIL), Instituto de Investigaciones Bioquímicas Buenos Aires (IIBBA) – Consejo Nacional de Investigaciones Científicas y Técnicas (CONICET), C1405BWE Buenos Aires, Argentina

## Abstract

Plants are plastic organisms that optimize growth in response to a changing environment. This adaptive capability is regulated by external cues, including light, which provides vital information about the habitat. Phytochrome photoreceptors detect far-red light, indicative of nearby vegetation, and elicit the adaptive shade-avoidance syndrome (SAS), which is critical for plant survival. Plants exhibiting SAS are typically more elongated, with distinctive, small, narrow leaf blades. By applying SAS-inducing end-of-day far-red (EoD FR) treatments at different times during Arabidopsis (*Arabidopsis thaliana*) leaf 3 development, we have shown that SAS restricts leaf blade size through two distinct cellular strategies. Early SAS induction limits cell division, while later exposure limits cell expansion. This flexible strategy enables phytochromes to maintain control of leaf size through the proliferative and expansion phases of leaf growth. mRNAseq time course data, accessible through a community resource, coupled to a bioinformatics pipeline, identified pathways that underlie these dramatic changes in leaf growth. Phytochrome regulates a suite of major development pathways that control cell division, expansion, and cell fate. Further, phytochromes control cell proliferation through synchronous regulation of the cell cycle, DNA replication, DNA repair, and cytokinesis, and play an important role in sustaining ribosome biogenesis and translation throughout leaf development.

## Teaser

Phytochrome regulates leaf blade plasticity through two alternative cellular response strategies and concertedly coordinates gene expression of the basic cellular machinery of leaf development.

## Introduction

Plants are highly malleable organisms that are able to adjust their growth strategy to a changing environment. The leaf is an excellent example of a highly plastic organ, where shape and size are not predetermined, but influenced by external signals, such as light. These adaptative qualities are important for survival because leaves perform critical roles in temperature regulation, gas exchange, and sunlight capture for photosynthesis (Tsukaya, 2005; Fritz et al., 2018). Leaves initiate at the shoot apical meristem, in a process involving different axes of symmetry (proximo-distal, adaxial–abaxial, medio-lateral). The leaf lamina, or blade, grows to its final size through a series of partially overlapping phases including cell division, transition, meristemoid division, and cell expansion (Gonzalez et al., 2012). The final leaf size and shape are ultimately determined by the relative contribution of these developmental components (Gonzalez et al., 2012; Kalve et al., 2014; Fritz et al., 2018).

The plant’s surrounding light environment can be monitored by a set of light-sensing systems, which play important roles in driving adaptive growth (Krahmer et al., 2018; Legris et al., 2019). The family of red (R)/far-red (FR)-absorbing phytochromes (phyA-E) possess unique photochemical properties that enable the detection of vegetation habitats that have high levels of FR compared with R light wavelengths. Phytochromes exist in a dynamic equilibrium of two photoconvertible forms: an inactive R-absorbing form (Pr) and a biologically active FR-absorbing form (Pfr; Holmes and Smith, 1975; Smith, 1982). Red light wavelengths present in natural light photoconvert Pr to the active Pfr form, while FR switches phytochrome back to the inactive Pr state. The FR-rich conditions of vegetation shade shift the dynamic phy equilibrium toward the inactive Pr form, which initiates an adaptive response known as the shade-avoidance syndrome (SAS). It is principally the deactivation of phyB, and to a lesser extent, other, the so-called, light stable phys C-E, that drives the SAS (Carabelli et al., 1996; Franklin, 2008; Franklin and Quail, 2010). Though, in continuous FR, phyA, which is normally light labile, is activated, accumulates in the nucleus, and operates to suppress the SAS (Hiltbrunner et al., 2005; Strasser et al., 2010; Rausenberger et al., 2011). In Arabidopsis (*Arabidopsis thaliana*), the SAS is characterized by reduced biomass, elongated petioles, exaggerated leaf hyponasty, and smaller leaf blades (Reed et al., 1993; Franklin and Whitelam, 2005; Tsukaya, 2005; de Wit et al., 2015; Galvao and Fankhauser, 2015; Goyal et al., 2016; Yang et al., 2016). However, it is noteworthy that in different conditions, for example, cooler temperatures, the SAS can lead to an increase rather than a reduction in leaf area, so the physical features of this response are conditional (Robson et al., 1993; Devlin et al., 1999; Franklin et al., 2003; Patel et al., 2013). Interestingly, earlier work established that the SAS is mainly elicited in the evening due to circadian gating by the clock (Salter et al., 2003; Mizuno et al., 2015). This means that daily end-of-day FR (EoD FR) treatments that coincide with a permissive gating window are relatively effective in eliciting the SAS (Salter et al., 2003; Mizuno et al., 2015). Further, the application of a short pulse rather than a prolonged FR treatment avoids the activation of phyA, which can antagonize SAS (Strasser et al., 2010). Thus, while there are some limitations, EoD FR has been deployed as useful tool to interrogate the SAS (Nagatani et al., 1991; Devlin et al., 1999; Salter et al., 2003; Franklin, 2008).

There is a growing body of information on how the SAS alters the leaf petiole. Application of phy-deactivating FR light triggers rapid leaf hyponasty and promotes petiole elongation (Ballare and Scopel, 1997; Sasidharan et al., 2010; Casal, 2013; Dornbusch et al., 2014; Michaud et al., 2017). Transcriptome analyses have been particularly instructive in defining the key operational pathways in the SAS and have, for instance, uncovered a central role for auxin and identified auxin pathway components that control elongation and hyponasty (Kozuka et al., 2010; Pantazopoulou et al., 2017). Petiole cell elongation is mediated by local FR-induced auxin response. Meanwhile, hyponasty, which results from differential abaxial–adaxial cell growth at the base of the petiole, is perceived at the leaf tip and executed by local auxin synthesis followed by transport to the petiole (Michaud et al., 2017; Pantazopoulou et al., 2017).

Alongside petiole elongation and hyponasty, the SAS can drastically limit leaf blade growth. Less is known about how this response is regulated, though an earlier study which measured *CYCLINB1;1-GUS* (*CYCB1;1-GUS*) activity indicated that shade exposure curtails the duration of the leaf cell division phase (Carabelli et al., 2007). Further support for this notion comes from a more recent report showing a low R:FR light ratio induces earlier mesophyll cell differentiation, which is associated with *ARABIDOPSIS THALIANA HOMEOBOX PROTEIN 2 (ATHB-2)* control of cell cycling cessation (Carabelli et al., 2018). Contrasting with these reports, another study showed that leaf growth modulation by low R:FR light is primarily mediated by changes in cell expansion (Patel et al., 2013). We currently lack a definitive understanding that reconciles these two observations and only have limited information on how low R:FR shade influences the major leaf development pathways.

In this study, we used leaf 3 (L3) as a representative model, and since vegetation shading can occur at any point during the plants’ life cycle, we used EoD FR as a tool to deactivate phy at different times during leaf development. We have found EoD FR can restrict blade growth by limiting cell division or cell expansion, depending on the timing of the EoD FR signal. This effect is mainly dependent on phyB, with a smaller contribution from other light stable phys. Further, we performed the first SAS mRNAseq time series analysis for the leaf blade, which coupled to a stringent in-depth bioinformatics analysis pipeline allowed us to move beyond the current understanding, which is largely hormone focused, to identify previously unknown roles for phy in the temporal coordination of major leaf development pathways and basic cellular processes that are critical for cell division and protein translation. To ensure findability, accessibility, and reusability of our data, we created an interactive web application where the expression of genes of interest can be visualized (https://aromanowski.shinyapps.io/leafdev-app/).

## Results

### The *phyB* null mutant has reduced leaf blade cell number

**Figure 1:**
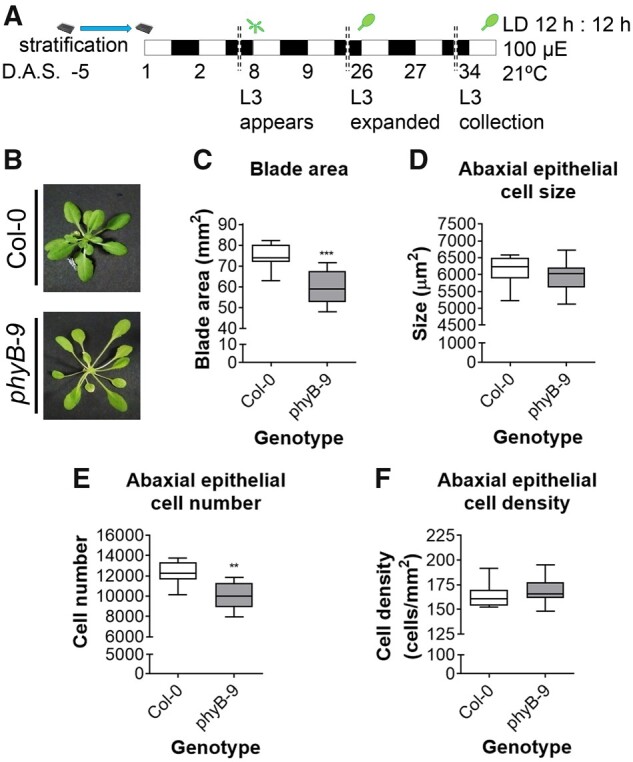
**Photoreceptor knockout mutants exhibit a diminished leaf blade area. A,** Schematic representation of the experimental conditions. The light blue arrow indicates the amount of time that the seeds have been stratified for in the trays. White rectangles indicate the 12 h of day period. Black rectangles indicate the 12 h of dark period. Doubled dashed lines indicate several days have passed in the same conditions. The plant drawn on top of Day 8 indicates L3 emergence. The leaf drawn on top of Day 26 indicates that L3 is fully expanded. The leaf on top of Day 34 indicates the time at which L3 has been collected. B, Pictures of rosettes of representative 34 D.A.S. *Arabidopsis thaliana* Col-0 wild type (top) and *phyB-9* mutant (bottom). C–F, Box plots of (C) blade area comparison (*n* = 12; Student’s *t* test; ***P < 0.001; GraphPad Prism); (D) abaxial epithelial cell size comparison (*n* = 360; Student’s *t* test; ns; GraphPad Prism); (E) abaxial epithelial cell number (*n* = 360; Student’s *t* test; **P < 0.01; GraphPad Prism); and (F) abaxial epithelial cell density (*n* = 12; Student’s *t* test; NS; GraphPad Prism). In all box plots: center line, median; box limits, 25–75th percentiles; whiskers, min to max; points, outliers. L3 = Leaf 3.

To establish the cellular basis for phyB control of leaf blade area, we measured leaf dimensions, abaxial epithelial cell number, cell size, and cell density parameters in fully expanded third leaves in the *phyB-9* mutant (see Figure 1A for growth regime schematic and Supplemental Figure 1, A–D for examples of leaf imprints). Leaf three (L3), which exhibits a qualitatively similar response to other leaves, was selected to aid comparison with other studies (Andriankaja et al., 2012; Woo et al., 2016). Consistent with the published data (Tsukaya et al., 2002), we observed a marked reduction (35.7%) in *phyB-9* leaf blade area compared with WT (Figure 1, B and C). We also found total cell number is reduced in *phyB-9*, while cell size and density are comparable with WT (Figure 1, D–F). To capture potential variation across the leaf, we compared cell size in the base, middle, and distal portions of the leaf blade. At each location, we established that cell number but not size was diminished in *phyB-9* (see Supplemental Figure 1, E and F), implicating phyB in the promotion of cell division within the leaf.

### Phytochrome control of L3 cellular response is developmental time dependent

**Figure 2:**
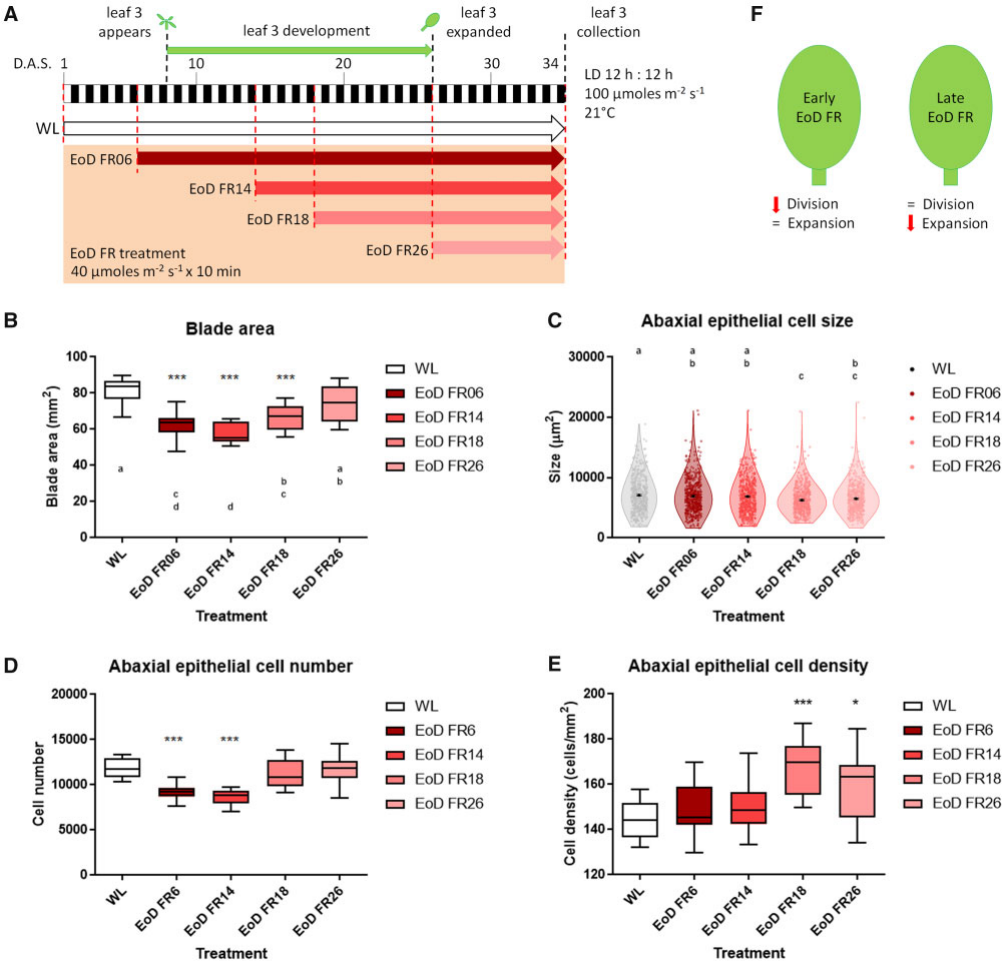
The effect of deactivating phytochromes is developmental timing regulated. A, Schematic representation of the experimental conditions. White rectangles indicate the 12 h of day period. Black rectangles indicate the 12 h of dark period. The green arrow indicates the period of L3 development, which is enclosed between two black-dashed lines. The plant drawn on top of Day 8 indicates L3 emergence. The leaf drawn on top of Day 26 indicates that L3 is fully expanded. The red-dashed lines indicate the day at which a specific treatment was started and coincides with a specific colored arrow (WL in white and EoD FR 06, 14, 18, and 26 treatments in decreasing tones of red, respectively). The red-dashed line at the end of Day 34 marks the end of each treatment. The black-dashed line on Day 34 indicates tissue collection. B, Box plot of L3 blade areas after each treatment (*n* = 12 leaves per condition; one-way ANOVA followed by Dunnett’s test; ***P < 0.001 versus WL; GraphPad Prism; means that do not share a letter are significantly different; Minitab). In the box plot: center line, median; box limits, 25–75th percentiles; whiskers, min to max; points, outliers. C, Violin and dot plots showing the distribution of cell sizes after each treatment (*n* = 2880 cells per condition; one-way ANOVA followed by Tukey’s multiple comparison test; means that do not share a letter are significantly different; Minitab). In the violin plot: center black dot, mean; error bars, Standard Error of the Mean (sem); violin limits, min to max. D, Box plot of total number of cells (*n* = 12 leaves per condition; one-way ANOVA followed by Dunnett’s test; ***P < 0.001 versus WL; GraphPad Prism). In the box plot: center line, median; box limits, 25–75th percentiles; whiskers, min to max; points, outliers. E, Box plot of epidermal cell density (*n* = 12 leaves per condition; one-way ANOVA followed by Dunnett’s test; *P < 0.05 and ***P < 0.001 versus WL; GraphPad Prism). In the box plot: center line, median; box limits, 25–75th percentiles; whiskers, min to max; points, outliers. F, Model depicting the strategy used by phytochrome deactivation in early or late treatment to reduce leaf blade area. L3 = Leaf 3; FR = far-red; EoD FR06 = EoD FR since Day 6; EoD FR14 = EoD FR since Day 14; EoD FR18 = EoD FR since Day 18; EoD FR26 = EoD FR since Day 26.

In nature, phyB deactivation by vegetation shading can occur at any point during leaf development. We, therefore, wanted to establish the developmental window in which phyB inactivation was most effective in limiting leaf growth. Here, we grew plants in standard 12:12 photoperiods with or without an EoD FR treatment which photoconverts phyB Pfr to its inactive Pr form (Supplemental Figure S2A). Previous studies have shown that EoD FR to a large extent is able to mimic the *phyB* null mutant phenotype (Johnson et al., 1994; Roig-Villanova and Martinez-Garcia, 2016). Concurring with these observations, plants exposed to daily EoD FR from Day 6 (prior to L3 emergence) until sampling on Day 34 exhibit a qualitatively similar response to *phyB-9*, with reductions in L3 blade area, and have lower cell number than controls (Supplemental Figure S2, B–E). However, it is worth noting that *phyB-9* plants treated with EoD FR still exhibit a small but significant decrease in cell number compared with those in standard conditions (Supplemental Figure S2D). This indicates that the reduced leaf blade area and cellular response are mainly dependent on phyB action and other light stable phytochromes contribute, but to a lesser extent. Next, we applied the same daily EoD FR regime but started the treatment at different times through L3 development (Days 6, 14, 18, or 26; Figure 2A). As expected, application of EoD FR early-on in leaf development through the cell division intense phase suppressed leaf blade expansion (Figure 2B). We also found that treatments from Day 18 were effective in repressing blade growth, albeit to a lesser extent (Figure 2B). Leaf blade size in the late EoD FR-treated population (from Day 26) overlapped significantly with white light (WL) but was more variable (Figure 2B). Treatments that commenced on Day 6 or 14 resulted in reductions in cell number, while EoD FR from Day 18, and to a lesser extent from Day 26 treatment, gave rise to reductions in cell size and corresponding increases in cell density (Figure 2, C–E). Col-0 plants carrying the CYCB1;1 promoter fused to the CYCB1;1 D-box-GUS/GFP construct further confirmed that early treatments affected cell division (Supplemental Figure S2F). These data indicate that phyB deactivation can reduce leaf blade expansion early in leaf development by imposing limits on cell division, or later by constraining cell expansion (Figure 2F).

### Gene expression profiling through leaf 3 developments

**Figure 3:**
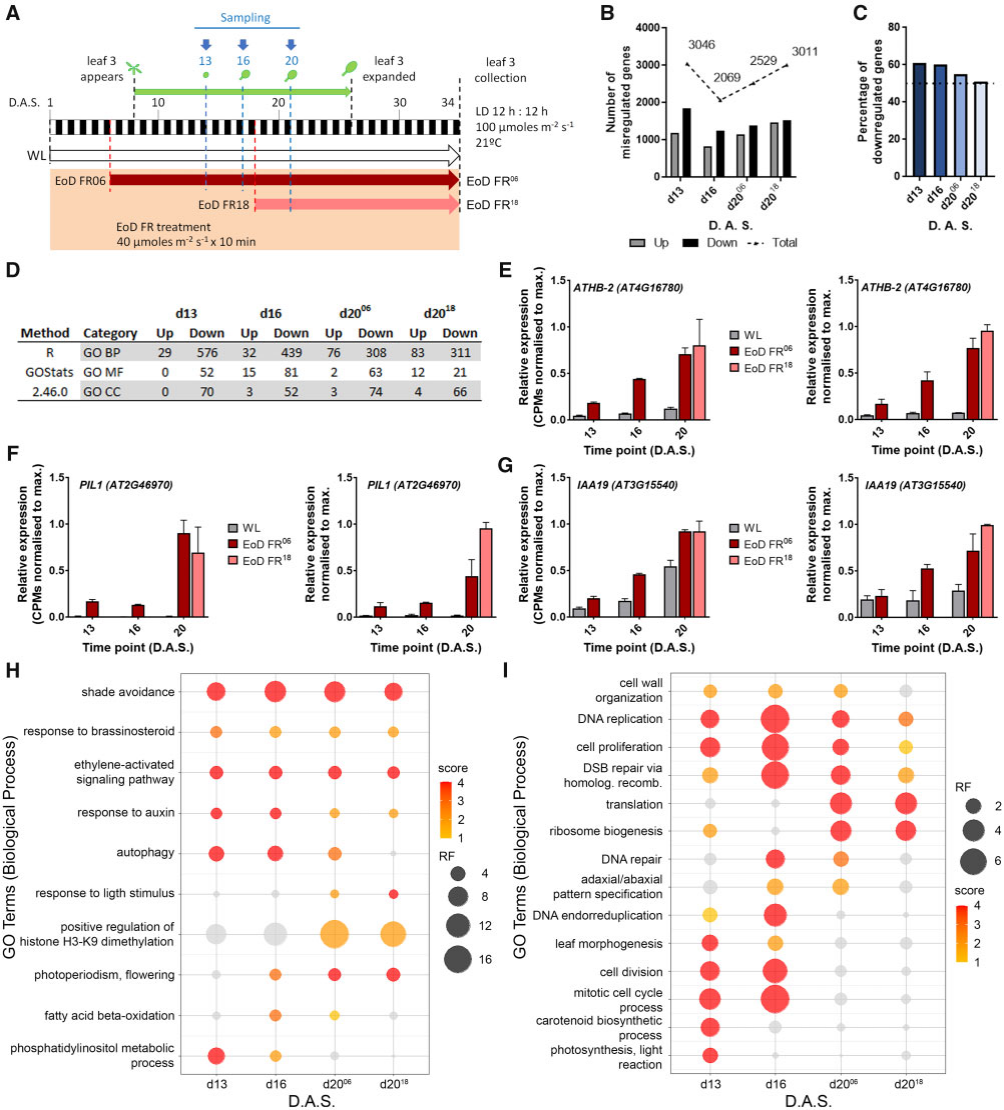
Early EoD FR treatment affects global gene expression mainly by downregulation. A, Schematic representation of the experimental conditions and sampling of Col-0 plants for the mRNAseq. White rectangles indicate the 12 h of day period. Black rectangles indicate the 12 h of dark period. The green arrow indicates the period of L3 development, which is enclosed between two black-dashed lines. The plant drawn on top of Day 8 indicates L3 emergence. The leaf drawn on top of Day 26 indicates that L3 is fully expanded. The red-dashed lines indicate the day at which a specific treatment was started and coincides with a specific colored arrow (WL in white and EoD FR 06 and 18 treatments in dark red and pink, respectively). The blue-dashed lines, blue arrows, and primordia (3 D.A.S.) and L3 (16 and 20 D.A.S.) drawings indicate the days at which tissue was collected. The black-dashed line at the end of day 34 marks the end of each treatment. B, Number of mis-regulated genes (differentially expressed genes) per time point (dark bars indicate up regulated genes, gray bars indicate downregulated genes, and dashed line indicates total number of mis-regulated genes). C, Time point analysis of the percentage of downregulated genes as compared with the total number of mis-regulated genes. The dotted line indicates 50%. D, Comparison of Gene Ontology (GO) Terms by time point, affected by downregulation or upregulation (*P* < 0.05 and *q* < 0.1; Hypergeometric Test with Benjamini-Hochberg correction; CC = CC). E–G, Normalized counts (left) and qPCR validation (right) of *ATHB-2* (E), *PIL1* (F), and *IAA19* (G) (*n* = 2 biological replicates with three technical replicates per time point per condition). Error bars represent S.E.M.; EoD FR^06^ = EoD FR since Day 6; EoD FR^18^ = EoD FR since Day 18. H, I, Bubble plot representation of a subset of processes up (H) or down (I) regulated by EoD FR treatment. Color represents *P* value score (1 = *P* < 0.05; 2 = *P* ≤0.01; 3 = *P* ≤0.001; 4 = *P* ≤0.0001; ns terms appear in gray color; Hypergeometric Test with Benjamini–Hochberg correction), and size of the bubble represents the representation factor (RF). L3 = Leaf 3; FR = far-red; d13 = day 13 under EoD FR since Day 6 treatment; d16 = Day 16 under EoD FR since Day 6 treatment; d20^06^ = Day 20 under EoD FR since Day 6 treatment; d20^18^ = Day 20 under EoD FR since Day 18 treatment.

As our data point to phytochrome control of both leaf cell proliferation and expansion phases, our next aim was to determine the underlying transcriptome regulation. Here, we exposed plants to either daily EoD FR from Day 6 (EoD-FR^06^), and harvested L3 primordia or blade tissue at ZT22 on Days 13, 16, and 20, or EoD FR from Day 18 (EoD-FR^18^), sampling at Day 20. Gene expression profiles were determined using Illumina mRNA sequencing (mRNAseq) (Figure 3A;Supplemental Table S1). Briefly, gene counts were extracted with the ASpli R package (Mancini et al., 2021). Raw counts were then filtered to remove weakly expressed genes, normalized to library size and expression was computed using EdgeR (Robinson et al., 2010), and the AtRTD2 annotation (Zhang et al., 2017). This resulted in 18,934 genes (55% of the 34,212 annotated AtRTD2 genes) to be considered for further downstream analysis. We then sought to assess the validity of our approach by examining the expression patterns of the known shade-induced genes, *ATHB-2*, *PHYTOCHROME INTERACTING FACTOR 3-LIKE 1 (PIL1), INDOLEACETIC ACID INDUCED 19 (IAA19), CYTOKININ OXIDASE 5 (CKX5), YUCCA 8 (YUC8), 1-AMINO-CYCLOPROPANE-1-CARBOXYLATE SYNTHASE 8 (ACS8)*, and the shade-repressed gene *TRP AMINOTRANSFERASE OF ARABIDOPSIS1 (TAA1*; Kozuka et al., 2010; Li et al., 2012; Pantazopoulou et al., 2017; Figure 3, E–G and Supplemental Figure S3). As determined by mRNAseq data, and qPCR we observed robust EoD FR responses for each of the FR shade-responsive genes (Figure 3, E–G;Supplemental Figure S3). Interestingly, all these genes responded robustly to EoD FR irrespective of when the treatment was applied during leaf development, revealing why these frequently studied marker genes are reliable reporters of SAS activation.

### EoD FR treatment favors downregulation rather than upregulation of biological processes

To gain further insights into the regulation of gene expression through L3 development, we developed custom R scripts to first perform a time point by time point differential gene expression (DGE) analysis using EdgeR, followed by gene ontology (GO) enrichment analysis and Kyoto Encyclopaedia of Genes and Genome (KEGG) pathway analysis. This approach, in contrast to previous studies that used a single developmental time point and were mainly focused on hormone responses (Kozuka et al., 2010; Pantazopoulou et al., 2017), allowed us to more broadly analyze gene expression changes throughout L3 development. Expression levels of individual genes (logCPM) and DGE profiles (log_2_FC) during the time course can be viewed at https://aromanowski.shinyapps.io/leafdev-app/. The mRNAseq data show that 28.5% (5,393/18,934; logFC > 0.58, *P* < 0.05 and *q* < 0.1) of all expressed genes were affected by EoD FR^06^ treatment at some point throughout L3 development, with 3,046, 2,069, and 2,529 genes mis-regulated at d13, d16, and 20, respectively (Figure 3B;Supplemental Figure S4 and Supplemental Table S2). A total of 3,011 mis-regulated genes was recorded at the EoD FR^18^ d20 time point, which was slightly higher than at EoD FR^06^ d20 (Figure 3B;Supplemental Figure S4 and Supplemental Table S2). A higher proportion of the EoD FR^0^^6^ category was downregulated, though this effect reduces with leaf age and is not seen in EoD FR^18^ d20 (Figure 3C). GO enrichment analysis established that processes from each of the three main GO categories – biological processes (BPs), molecular function (MF), and cellular component (CC) – were strongly overrepresented in the downregulated category (Figure 3D, full list of GO terms in Supplemental Table S3 and REVIGO summarization in Supplemental Table S4). A more stringent DGE analysis (logFC >0.58, *P* < 0.05 and *q* < 0.05) resulted in a lower number of mis-regulated genes but did not qualitatively alter these observations (Supplemental Figure S5).

The smaller number of upregulated processes included BP categories that have been previously studied, such as shade avoidance, autophagy, response to hormone signaling pathways, or flowering (Nozue et al., 2015; Pantazopoulou et al., 2017; Kim et al., 2018). Shade avoidance and auxin and ethylene signaling are upregulated at all time points, while autophagy is more upregulated in earlier time points, and flowering later-on (see bubble plot on Figure 3H, where the size of the circle represents the representation factor (RF) and the color indicates the *P*-value score, and Supplemental Table S3). Likewise, as expected, photosynthesis and carotenoid biosynthesis are downregulated by EoD FR, but only on d13. However, the most significantly downregulated category groups include mitotic cell cycle and other associated processes such as cell proliferation, cell division, and DNA replication, DNA repair, and DSB repair (Figure 3I;Supplemental Table S3). For all these processes, the repressive effect of EoD FR was most severe on d16. This analysis also illustrates that ribosome biogenesis and translation are strongly suppressed by EoD FR, but only later-on in leaf development. These data implicate phyB as a key regulator of multiple processes involved in cell proliferation in the leaf. Further, they identify processes not previously known to be phyB-regulated, such as DNA repair and ribosome biogenesis.

### EoD FR-activated transcription factors and hormone signaling pathways

**Figure 4:**
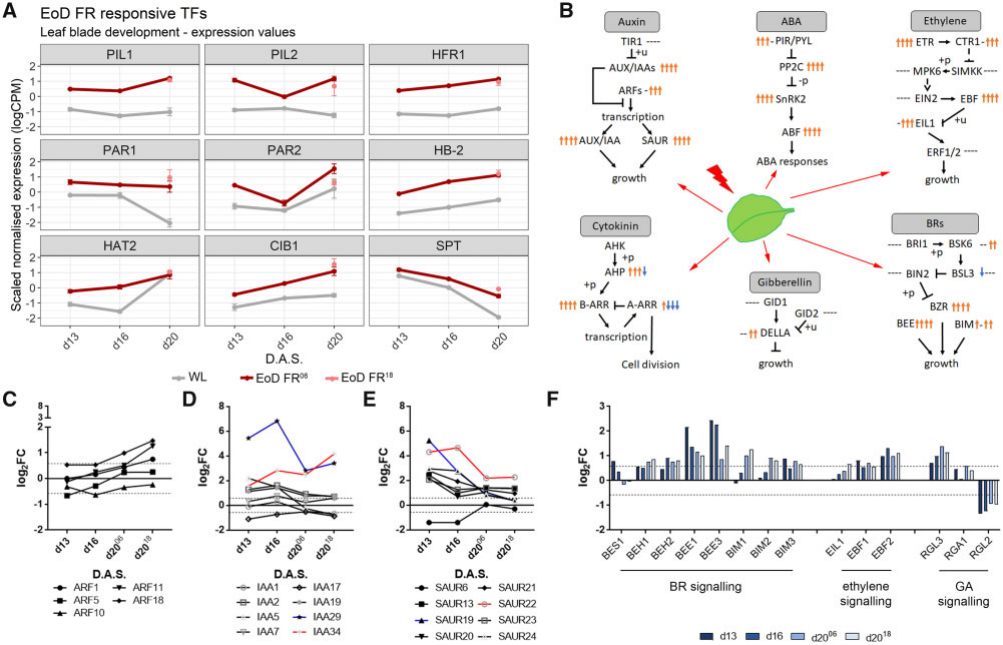
Shade responsive transcription factors and hormone pathways affected by EoD FR treatment. A, Gene plots of TFs affected by EoD FR under WL (gray), EoD FR^06^ treatment (dark red), and EoD FR^18^ treatment (pink) across all time points (error bars indicate sem). B, Schematic representation of different hormone signaling pathways and their components affected by EoD FR (red-lightning bolt). Simplified diagrams for auxin, ABA, ethylene, BR, GA, and cytokinin signaling with average component expressions across each time point shown (adapted from the KEGG metabolic pathway analysis). Orange arrows indicate upregulation, blue arrows indicate downregulation, and ‘-’ indicate no significant changes from WL conditions. Symbols are arranged from left to right representing d13, d16, d20^06^, and d20^18^, respectively. ‘−/+p’ and ‘+u’ indicate de/phosphorylation and ubiquitination, respectively. C–E, Line plots of log_2_FC DGE values of auxin signaling components ARFs (C), IAAs (D), and Clade II SAUR (E) genes affected by EoD FR, compared with WL conditions. *IAA29* (blue line) and *IAA34* (red line) are the highest upregulated IAAs (D) *SAUR19* (blue line) and *SAUR22* (red line) are the highest upregulated SAURs (E). The dashed lines indicate the |log_2_FC| = 0.58 threshold. F, Bar plots of log_2_FC DGE values of BR, ethylene, and GA (from left to right) downstream signaling genes affected by EoD FR, compared with WL conditions. The dashed lines indicate the |log_2_FC| = 0.58 threshold; FR = far-red; d13 = Day 13 under EoD FR since Day 6 treatment; d16 = Day 16 under EoD FR since Day 6 treatment; d20^06^ = Day 20 under EoD FR since Day 6 treatment; d20^18^ = Day 20 under EoD FR since Day 18 treatment.

Among the transcription factors (TFs) most highly regulated by EoD FR are classical shade response genes, such as *PIL1, PIL2, HFR1, PAR1, PAR2, ATHB-2*, and *HAT2* (Figure 4A;Supplemental Table S5). Several B-BOX genes (*BBX6, 17, 21, 23, 27, 28, and 29*) are upregulated, as are 8 *NUCLEAR FACTOR-Y (NF-Y)* genes, previously shown to complex with some BBX’s (Myers et al., 2016; Gnesutta et al., 2017; Supplemental Figure 6A;Supplemental Table S5). Also upregulated are the *CRY2/CIB5* interacting partner *CIB1* (Liu et al., 2008; Liu et al., 2013); and *SPT*, involved in flowering, temperature, and shade-dependent growth promotion (Sidaway-Lee et al., 2010; Nozue et al., 2015; Wu et al., 2018; Figure 4A;Supplemental Table S5). Notably, the majority of these shade response TFs are upregulated by EoD FR at all time points.

A general outline for the main plant hormone signaling pathways, as determined by KEGG pathway analysis, can be seen in Figure 4B. The data show that EoD FR mainly leads to the upregulation of hormone signaling (Supplemental Table S2). This finding was confirmed by hormonometer analysis (Volodarsky et al., 2009), which found strong transcriptome signatures for auxin, ethylene, and abscisic acid (ABA) at all time points, and for gibberellin (GA), brassinosteroid (BR), and cytokinin early on (Supplemental Figure S6B). For auxin pathway genes, there is a tendency for expression of *ARFs* to increase with successive EoD FR treatments, while for most *IAA*, the earlier EoD FR treatments were the most effective (Figure 4, C and D). Of these genes, *IAA29 and IAA34* are among the most highly expressed. The response of *SMALL AUXIN UP-REGULATED RNA (SAUR)* genes fell into two main subsets: one that gradually increased their expression and another with the opposite regulation (Figure 4E;Supplemental Figure S6C). BR genes *BZR*, *BEE1, BEE3, BIM1-3, BES1*, and *BEH1-2* and ethylene synthesis (*ACS8*) and signaling genes *EIL1* and *EBFs* are upregulated by EoD FR. The GA-responsive, growth-supressing DELLA genes *RGL3* and *RGA1* are upregulated by EoD FR, while *RGL2* expression is repressed (Figure 4F;Supplemental Table S2). In the case of cytokinin signaling, EoD FR promotes the expression of *AHP5*, and particularly *AHP1* on d13. Most type of B-ARRs is upregulated by early EoD FR exposure, while the type A-ARRs *ARR4* and A*RR16* exhibit gradual suppression by sequential EoD FR (Supplemental Figure S6D). Finally, with a few exceptions, different classes of ABA signaling genes are upregulated by EoD FR, including PP2C (*ABI1* and *HAI1*), *ABF* genes (*ABI5, EEL*, and *ABF3 and ABF4)*, SnRK2 genes (*SNRK2.2, SNRK2.3, SNRK2.5*, and *SNRK2-8*), and *PYR/PIL* genes (*RCAR1, RCAR3*, and *PYL7*; Supplemental Figure S6E).

### EoD FR treatment suppresses basic cellular processes required for leaf cell division

**Figure 5:**
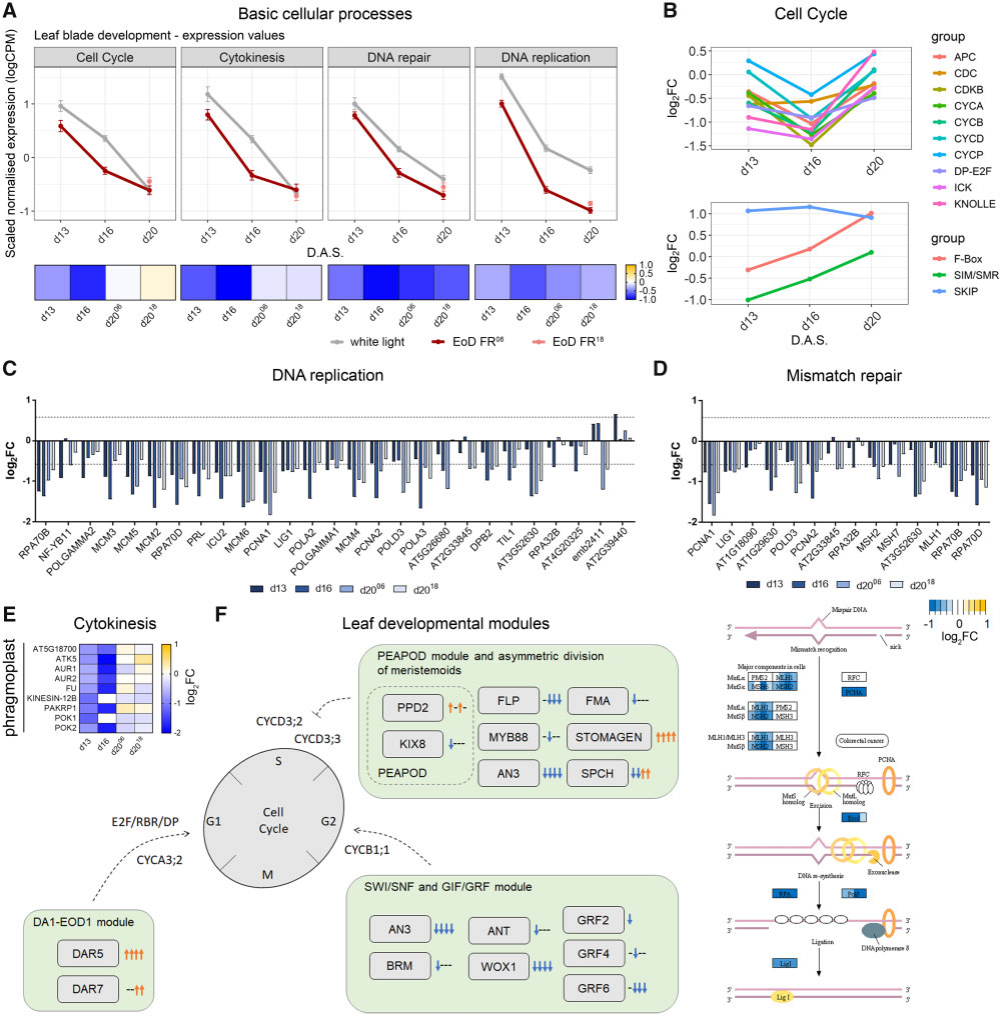
Basic cellular processes and leaf development modules affected by EoD FR treatment. A, Mean expression of differentially expressed cell cycle, cytokinesis, DNA repair, and DNA replication genes under WL (gray), EoD FR^06^ treatment (dark red), and EoD FR^18^ treatment (pink) across all time points (error bars indicate sem). The heatmap below each line graph indicates the average log_2_FC values of genes involved in each process. d13 = Day 13 under EoD FR since Day 6 treatment; d16 = Day 16 under EoD FR since Day 6 treatment; d20^06^ = Day 20 under EoD FR since Day 6 treatment; d20^18^ = Day 20 under EoD FR since Day 18 treatment. B, Mean log_2_FC differential expression values of families of cell cycle regulators. (Top) APC, CDC, CDKB, CYCA, CYCB, CYCD, CYCP, DP-E2F, ICK, and KNOLLE genes. (Bottom) F-Box, SIM/SMR, and SKIP genes. Values correspond to samples under EoD FR since day 6 treatment, compared with WL conditions. C, D, Bar plots of DNA replication (C) and mismatch repair (D) genes affected by EoD FR. The dashed lines indicate the |log_2_FC| = 0.58 threshold. A modified schematic of the Mismatch Repair KEGG metabolic pathway from *A. thaliana* (ath03430) can be seen below the bar plot in (D). Original KEGG Graph data were (Kanehisa and Goto, 2000) rendered with the Pathview R package (Luo and Brouwer, 2013). E, Heatmap of log_2_FC values of genes involved in cytokinesis. d13 = Day 13 under EoD FR since Day 6 treatment; d16 = Day 16 under EoD FR since Day 6 treatment; d20^06^ = Day 20 under EoD FR since Day 6 treatment; d20^18^ = Day 20 under EoD FR since Day 18 treatment. F, Schematic representation of the different leaf developmental modules and their components affected by EoD FR. Simplified diagrams and their connections to the leaf cell cycle are shown, as described before (Vercruysse et al., 2020). Orange arrows indicate upregulation, blue arrows indicate downregulation, and ‘−’ indicate no significant changes from WL conditions. Symbols are arranged from left to right representing d13, d16, d20^06^, and d20^18^, respectively. FR = far-red; d13 = Day 13 under EoD FR since Day 6 treatment; d16 = Day 16 under EoD FR since Day 6 treatment; d20^06^ = Day 20 under EoD FR since Day 6 treatment; d20^18^ = Day 20 under EoD FR since Day 18 treatment.

The leaf blade cellular response data show that cell division is the major process that affects SAS leaf development when EoD FR is applied early in development (Figure 2; Supplemental Figure S2). A previous study showed that simulated shade leads to the early termination of leaf expressed *CYCLIN B1;1-GUS* (*CYCB1;1-GUS*; Carabelli et al., 2007, 2018). Our data concur with this observation but show that phyB has a much broader role in controlling cell division (Supplemental Figure S2F). Deactivation of phyB simultaneously represses genes that control cell cycle, DNA replication, DNA repair processes, and cytokinesis—all vital components of cell division (Figure 5A;Supplemental Tables S4 and S6). The expression of genes in each of these categories is high during the proliferative phase of leaf development (d13), falling gradually as the leaf matures. For cell cycle and cytokinesis genes, EoD FR treatment reduces expression on d13 and d16, which suppresses and potentially limits the duration of these processes. In contrast, DNA replication and repair are repressed by EoD FR throughout leaf development. Data in each of these categories are summarized below:

(1). Cell cycle: In plants, progression through the cell cycle is controlled by the CYCLINS (CYCs) complexed with CYCLIN-DEPENDENT KINASES (CDKs), the E2F/DIMERISATION PROTEIN (DP) transcriptional regulatory proteins, KIP-RELATED PROTEIN/INTERACTOR OF CDKs (KRP/ICK), and SIAMESE/SIAMESE-RELATED (SIM/SMR) proteins. EoD FR treatment suppresses the expression of genes in each of these categories, particularly on d16 (Figure 5B). Additional affected cell cycle regulators include *DP-E2F-like protein 3 (DEL3)* and *ETG1*, an E2Fa-DPa target (Figure 5B and Supplemental Table S2). CDK–CYC complexes are also regulated by proteolysis, which is mediated by the anaphase-promoting complex/cyclosome (APC/C) and the SKP1/CULLIN1/F-BOX PROTEIN complexes. Interestingly, F-box *SKP1 interacting partner 1 (SKIP1)*, *SKP2A*, and *SKP2B* are upregulated by EoD FR at all time points (Figure 5B;Supplemental Table S2).

(2). DNA replication and repair: Multiple genes controlling DNA replication are downregulated by EoD FR, including DNA polymerase α-primase, δ, and ε complexes; RPA; clamp (PCNA) and clamp loader (RFCs); the flap endonuclease (5'–3' exonuclease, AT5G26680); and DNA ligase. The minichromosome maintenance protein complex (MCM) DNA helicase is essential for genomic DNA replication. It is therefore notable that EoD FR suppresses the expression of all *MCM* genes (Figure 5C;Supplemental Table S2). DNA repair mechanisms are also strongly suppressed by EoD FR throughout L3 development. KEGG pathway visualization and DGE analysis revealed repression of genes encoding key enzymes in non-homologous end-joining, homologous recombination (HR)/homology-directed repair, nucleotide excision repair (NER), and base excision repair (BER; Supplemental Figure S7 and Supplemental Table S2). Remarkably, all the enzymatic steps in mismatch repair (MMR) are suppressed (Figure 5D;Supplemental Table S2).

(3). Cytokinesis: EoD FR downregulates cytokinesis genes specifically in early- and mid-leaf development. These genes include serine/threonine kinases *AURORA1 (AUR1)* and *AUR2; PHRAGMOPLASTIN-INTERACTING PROTEIN 1 (PHIP1);* microtubule end-binding proteins *EB1A*, *EB1B*, and *EB1C*; kinesins *HINKEL (HIK)* and *TETRASPORE (TES)*; ARM domain containing protein kinases *FUSED (FU)* and *RUNKEL (RUK/AT5G18700)*; Arabidopsis homolog of maize *TANGLED1* (*ATN);* and several microtubule-associated proteins, including kinesins such as *ATK5* and *KINESIN 12 (KIN12)* family members *PHRAGMOPLAST-ASSOCIATED KINESIN-RELATED PROTEIN 1 (PAKRP1), KIN12B, PHRAGMOPLAST ORIENTING KINESIN 1 (POK1)* and *POK2*; and spindle checkpoint proteins *BUB3.1* and *BUB3.2*. The developmental phase-specific regulation of these genes by EoD FR strongly corresponds with that for cell cycle genes (Figure 5E;Supplemental Table S2).

These data show phyB appears to have a broad operational role in regulating multiple processes involved in cell proliferation. Exposure to EoD FR from early leaf development suppresses DNA replication and repair, and appears to dampen and shorten the phase of cell cycle and cytokinesis gene expression.

### EoD FR controls the expression of key developmental pathways that control leaf cell proliferation

Our data provide evidence that EoD FR controls several key leaf development modules with connections to the leaf cell cycle, as follows (Figure 5F):

*AN3/GRF-SWI/SNF module:* EoD FR represses the expression of core members of the *AN3/GRF-SWI/SNF* module genes that are known to promote *CYCB1;1* expression and leaf cell proliferation (Vercruyssen et al., 2014; Vercruysse et al., 2020). EoD FR-suppressed genes include the central modulator, *ANGUSTIFOLIA 3/GRF INTERACTING FACTOR 1 (AN3/GIF1)*, as well as *GROWTH REGULATING FACTOR 2 (GRF2)*, *GRF4*, *GRF6*, *BRAHMA (BRM)*, *AINTEGUMENTA (ANT)*, and *WOX1/STF* (Figure 5F;Supplemental Table S2).

*DA1-EOD1 module*: *DA1* is a ubiquitin receptor that is proposed to operate with the E3 ligase *ENCANDER OF DA1-1/BIG BROTHER (EOD1/BB)* to restrict the duration of leaf cell proliferation and modulate the transition to endoreduplication by indirectly affecting the expression of the cell cycle genes *RETINOBLASTOMA RELATED (RBR)* and *CYCA3;2* (Peng et al., 2015; Vanhaeren et al., 2017). Further, *SUPPRESSOR OF DA1-1* (*SOD7, AT3G11580*) has also been shown to negatively regulate seed and leaf size (Zhang et al., 2015). Our data show the DA1 homolog *DA1-RELATED PROTEIN 5 (DAR5)* is upregulated at all time points, while *DAR7* is upregulated at d20 by EoD FR (Figure 5F;Supplemental Table S2). Further, EoD FR substantially upregulates the expression of *SOD7* later in the development (Supplemental Table S2). The HD-Zip II gene *ATHB-2* has been implicated in shade-induced early exit from cell proliferation in Arabidopsis leaves one and two (Carabelli et al., 2018). We note *ATHB-2* and its homologue *HAT2* are upregulated in EoD FR-treated L3 (Supplemental Table S2). EoD FR therefore may restrict the duration of leaf cell proliferation, partly by modulating *DA1-EOD1* module *and HD-Zip II* components*.* We also noted EoD FR upregulation of HD-Zip class Iβ genes *ATHB52* (all time points), *ATHB1* (d16 and d20), *ATHB6* (d16), and *ATHB16* (d16). Of these, ATHB16 has previously been shown to negatively regulate leaf cell expansion (Wang et al., 2003; Henriksson et al., 2005; Supplemental Table S2). Further, *BIG BROTHER (BB)*, whose overexpression leads to reduced cell size (Disch et al., 2006), is only upregulated on d20 of EOD FR^18^ (Supplemental Table S2).

*PEAPOD module and asymmetric division of meristemoids:* Almost half of the pavement cells in Arabidopsis leaves are the result of asymmetric divisions of meristemoids. *PEAPOD 2 (PPD2)*, a negative regulator of meristemoid asymmetric division which has been shown to directly bind to the *CYCD3;2* and *CYCD3;3* promoters to repress their transcription (Gonzalez et al., 2015), is slightly upregulated on d13 and d20 of EoD FR^06^ (Figure 5F;Supplemental Table S2). EoD FR downregulates genes involved in the sequential steps in guard cell formation. These include *SPEECHLESS (SPCH*; down on d13 and d16), which promotes asymmetric meristemoid division; *FOUR LIPS (FLP/MYB124*; down on d16 and d20), which controls symmetric division of mother guard cells; and *FAMA (FMA*; down on d20), which regulates guard cell formation (Lai et al., 2005; Ohashi-Ito and Bergmann, 2006; Lau et al., 2014). On the other hand, we found that *STOMAGEN (STOM)*, a mesophyll-expressed regulator of stomatal development, is upregulated at all time points (Sugano et al., 2010; Figure 5F;Supplemental Table S2).

*Adaxial-abaxial patterning:* Our data show downregulation of the adaxial fate development genes *PHABULOSA (PHB), ASYMMETRIC LEAVES 1 (AS1), AS2*, and the abaxial fate gene *KANADI 2 (KAN2)*, a homolog of KAN1 that has been linked to strong suppression of shade-avoidance responses (Xie et al., 2015), and *AINTEGUMENTA (ANT*; Supplemental Figure S8 and Supplemental Table S2). *BLADE ON PETIOLE 1* (*BOP1*) and *BOP2*, implicated in the control of adaxial–abaxial polarity genes and lateral organ fate, are also repressed by EoD FR (Ha et al., 2007; Supplemental Table S2).

Overall, our data point to a central role for phy in controlling developmental pathways that regulate leaf cell fate, cell proliferation/expansion, meristemoid cell division, which act in concert to determine overall leaf blade shape and size.

### EoD FR regulates ribosome biogenesis and translation later in leaf development

**Figure 6:**
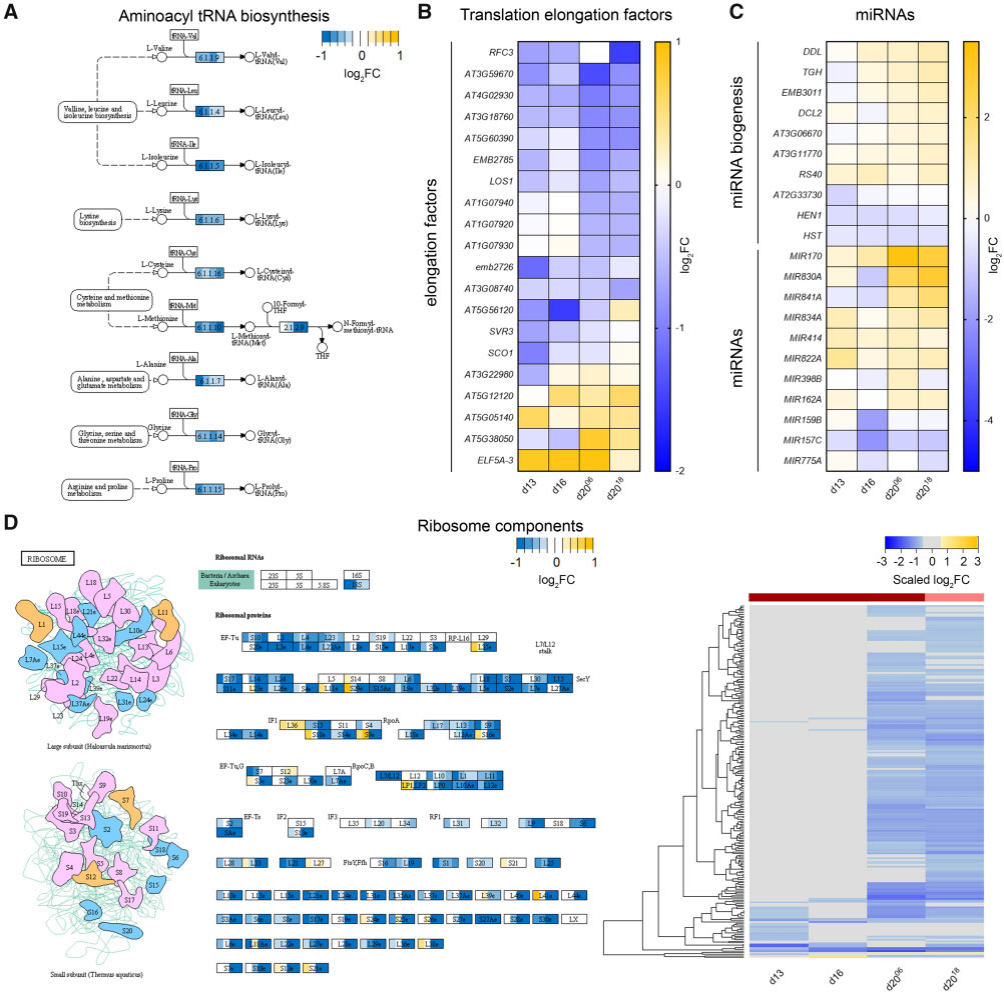
EoD FR affects known processes involved in translation. A, Modified schematic of the aminoacyl-tRNA biosynthesis KEGG metabolic pathway from *A. thaliana* (ath00970). Original KEGG Graph data (Kanehisa and Goto, 2000) were rendered with the Pathview R package (Luo and Brouwer, 2013). Dashed lines indicate an indirect link or unknown reaction, and solid lines indicate a molecular interaction or relation. Each rectangle is divided into four color regions reflecting the scaled logFC value of each time point. The regions are arranged from left to right representing d13, d16, d20^06^, and d20^18^, respectively. B, C, Heatmap of log_2_FC DGE data of elongation factor- (B) and miRNA biogenesis- and miRNA-(C) coding genes affected by EoD FR treatments. D, Modified schematics of the ribosome KEGG metabolic pathway from *A. thaliana* (ath03010; left) and heatmap of log_2_FC DGE data of ribosome component genes (right). Original KEGG Graph data (Kanehisa and Goto, 2000) were rendered with the Pathview R package (Luo and Brouwer, 2013). Each rectangle is divided into four color regions reflecting the scaled logFC value of each time point. The regions are arranged from left to right representing d13, d16, d20^06^, and d20^18^, respectively. The color bands above the heatmap indicate samples under EoD FR^06^ (dark red) or EoD FR^18^ (pink) treatment. In the heatmaps, d13 = Day 13 under EoD FR since Day 6 treatment; d16 = Day 16 under EoD FR since Day 6 treatment; d20^06^ = Day 20 under EoD FR since Day 6 treatment; d20^18^ = Day 20 under EoD FR since Day 18 treatment.

Alongside the suppression of leaf growth regulators, we also detected a strong EoD FR repression of translational processes (Supplemental Tables S3, S4, and S6). EoD FR application downregulates aminoacyl-tRNA synthetases throughout leaf development. The strongest repressive effects can be seen on genes involved in the Valine, Leucine, Isoleucine, Lysine, Cysteine, Methionine, Glycine, Proline, and Alanine aminoacyl-tRNA biosynthetic pathways (Figure 6A;Supplemental Table S2). We established that a high proportion of elongation factors directly involved in translation are suppressed by EoD FR, but only later in L3 development (Figure 6B). This late-phase timing coincides with upregulation of several miRNA biogenesis genes, such as *DICER-LIKE 2 (DCL2), DAWDLE (DDL)*, and *TOUGH (TGH)*, which are involved in the cleavage of pri- and pre-miRNAs (Moturu et al., 2020); and miRNA-coding genes (*MIR170, MIR830A, MIR841A, MIR834A, MIR414, MIR822A*, and *MIR162A*; Figure 6C). Interestingly, *MIR398B* targets the chaperone (*CCS1*), which is essential for protein maturation (Bouche, 2010). Perhaps, a most striking observation is the severe and coordinated repression of genes involved in ribosome biogenesis and genes coding for subunits of both the large and small ribosome complexes, again, later in L3 development (Figure 6D;Supplemental Figure S9 and Supplemental Table S2). Taken together, these data reveal that phyB deactivation by EoD FR has a profound repressive effect on ribosomes and basic translational processes, particularly in late leaf development.

## Discussion

### The SAS leaf response exhibits cellular response plasticity

Although genetic factors determine the blueprint of a leaf, environmental cues can have a pronounced effect on its final size. At high vegetation densities, shade-intolerant plants switch to a SAS survival growth strategy that reconfigures overall leaf architecture and is typified by a dramatic reduction in leaf blade area and elongated and hyponastic petioles (Legris et al., 2019). While the cellular responses and associated changes in the transcriptome have been elucidated for SAS petioles (Sasidharan et al., 2010; Michaud et al., 2017; Pantazopoulou et al., 2017; Legris et al., 2019), less is known of how the SAS influences leaf blade development. Published studies suggest the SAS limits blade growth by shortening the phase of leaf cell proliferation (Carabelli et al., 2007; Carabelli et al., 2018), while another study implicates cell expansion as a controlling factor (Patel et al., 2013). By introducing daily phy deactivating EoD FR at different times during leaf development, we were able to demonstrate that phys, mainly through phyB (Supplemental Figure S2), can control blade size by regulating cell division or expansion, depending on the developmental phase of the leaf (Figure 2). Early exposure to EoD FR limits cell division, while later exposure limits cell expansion. The ability to control both cell division and expansion phases enables phys to exert control on leaf growth throughout leaf development via alternative cellular processes.

### Deactivating phy with EoD FR leads to the widespread suppression of BP

Transcriptome studies have been instrumental in providing a system level understanding of how the SAS operates in the seedling and petiole (Sessa et al., 2005; Tao et al., 2008; Kozuka et al., 2010; Hornitschek et al., 2012; Li et al., 2012; Ciolfi et al., 2013; Pantazopoulou et al., 2017; Kim et al., 2018). These studies have identified important SAS markers and signaling pathways and have provided a critical understanding of the central role of hormones such as auxin. Furthermore, two of these studies analyzed leaf blade transcriptomes at a single discrete time point, mainly focusing on hormone responses (Kozuka et al., 2010; Pantazopoulou et al., 2017). Our study aimed to extend these insights by conducting an mRNAseq of L3 development (accessible online at https://aromanowski.shinyapps.io/leafdev-app/) coupled to an in-depth bioinformatics analysis pipeline. We showed that EoD FR led to the mis-regulation of 33.6% (6,357/18,934) of all detected expressed genes, when considering all sampled time points (Figure 3, B and C; Supplemental Figure S4, A and B, and Supplemental Table S2). Classic shade-responsive genes, such as *ATHB-2, PIL1, PIL2, PAR1, PAR2, HFR1. and HAT2*, were found to be reliably upregulated at all time points (Figures 3, E, F and 4, A; Supplemental Table S2). Our data indicated, however, that more genes were downregulated by EoD FR, particularly at the earlier sampling times (Figure 3, B and C; Supplemental Figure S4). Further, GO term analysis revealed that throughout leaf development, a very sizeable majority of processes were repressed by EoD FR (Figure 3D). These results are interesting in light of the overwhelming focus to date, on genes that are upregulated in SAS (Ballare and Pierik, 2017; Iglesias et al., 2018; Sessa et al., 2018). Indeed, the implication is that deactivation of phyB has a broadly repressive effect on BPs with wide-ranging roles in leaf growth and development.

### Phys-reprogrammed hormone signaling in the leaf

Consistent with previous studies, we observed EoD FR-induced changes in hormone signaling, particularly auxin, BR, ethylene, cytokinin, and ABA (Figure 4B). These were further confirmed by hormonometer analysis (Supplemental Figure S6B; Volodarsky et al., 2009), which found strong hormone transcriptome signatures for auxin, ethylene, and ABA throughout L3 development, and BR, GA, and cytokinin at earlier time points. Interestingly, these results closely matched the hormonometer analysis of an earlier study where plants were grown under short-day conditions and subjected to low R:FR ratio (R:FR = 0.05, fifth youngest leaves of 28-d-old plants; Pantazopoulou et al., 2017). The ‘leaf tip – whole FR’ dataset results of that study, which is the closest to our conditions, were highly similar to our d16 EoD FR-treated hormone signatures. Furthermore, similar transcriptome responses were found in another study where plants were grown for 19 d in continuous light and then subjected to a FR pulse followed by 2-h darkness (Kozuka et al., 2010). It is interesting to note that the action of several phytohormones, such as cytokinin, GA, auxin, and BR, is known to be involved with the leaf expansion process (Du et al., 2018; Ali et al., 2020). Under shade-like conditions, auxin, GA, BR, and ethylene have been associated with hypocotyl and petiole growth (Yang and Li, 2017). However, a study performed with leaf primordia exposed to canopy shade found a role for auxin-induced cytokinin oxidase in the repression of cell proliferation (Carabelli et al., 2007). In agreement with this, our data show EoD FR induction of *CKX5* at the primordia stage, but also throughout L3 development (Supplemental Table S2). Further studies will be required to gain a more comprehensive understanding of these pathways in SAS repression of leaf blade cell proliferation.

Previous transcriptomics studies have largely focused on a single discrete time point, while our time series approach allowed us to observe how the hormone responses changed throughout L3 development. For example, we observed opposing temporal regulation of several ARFs and IAAs by EoD FR. A large group of SMALL AUXIN UP-REGULATED RNA (SAUR) genes exhibited progressive upregulation by EoD FR throughout time, while a subset was sequentially downregulated. Among the latter, EoD FR specifically enhanced the expression of clade II (*SAUR13, SAUR19-24*) and clade IV SAUR genes (*SAUR63-67*; Kodaira et al., 2011; Stortenbeker and Bemer, 2019) at earlier time points in L3 development. Overall, our data suggest that early hormone signaling components are enhanced by EoD FR and that hormonal responses are quite nuanced with changes throughout L3 development.

### Phys are master regulators of cell proliferation

Earlier studies showed for the first two rosette leaves, persistent canopy shade restricts the period of CYCB1;1 expression (Carabelli et al., 2007, 2018). Our L3 mRNAseq data concur with this finding but show phys have a wider role, controlling multiple processes involved in cell division, including cell cycle, DNA replication, DNA repair, and cytokinesis. A previous report showed that genes involved in DNA synthesis, DNA repair, and cell cycle were among a cluster of 3,817 genes that were normally switched on early in L3 development and then progressively switched off during the transition from proliferative to cell expansion phase (Andriankaja et al., 2012). This trend is clearly seen in our L3 mRNAseq data (Figure 5A), but so is the impact of EoD FR on dampening these processes (Figure 5A–E and Supplemental Figure S7).

The progression of the cell cycle is tightly regulated by core cell cycle protein CYCs complexed with CDKs, the E2F-DP heterodimer (which transcriptionally regulate cell cycle machinery genes), and the cell cycle inhibitor proteins KRP/ICK and SIM/SMR. Specific CDKs bind with different CYC types (CYCA, CYCB, CYCD, CYCP) to control different transition points through the cell cycle (Vercruysse et al., 2020). Deactivation of phyB-E with EoD FR represses the expression of *CDKB, CYCA, CYCB, CYCD*, and *CDC* genes, implicating these phys in controlling multiple steps in the cell cycle. EoD FR suppresses the expression of the DP factor *DEL3*, the E2Fa-DPa target *ETG1*, and the CDK inhibitors *ICK5* and *ICK6*. Simultaneously, EoD FR leads to upregulation of *SKIP1, SKP2A*, and *SKP2B*, which are involved in the proteolysis of CDC/CYC complexes. Overall, our data indicate that phy exerts a strong influence on cell cycle progression during the early phase of L3 development.

DNA replication and repair are downregulated by EoD FR throughout L3 development. Here, we observe the repression of essential DNA replication components, including the DNA polymerase α-primase, δ, and ε complexes; the MCM complex; RPA; clamp (PCNA) and clamp loader (RFCs); the flap endonuclease (5'–3' exonuclease, AT5G26680); and DNA ligase (Figure 5C). To ensure that DNA replication is error-free, plants utilize several DNA repair mechanisms, which are coupled to cell division and the cell cycle (Branzei and Foiani, 2008; Manova and Gruszka, 2015). Daily deactivation of phyB leads to suppression of the HR, NER, BER, and MMR DNA repair pathways, with the strongest effect observed for MMR type DNA repair (Figure 5D;Supplemental Figure S7). In yeasts, MMR factors work in concert with the replication machinery to repair errors that occur in the daughter strand shortly after replication, hence increasing its fidelity (Jiricny, 2013).

Our analysis has also revealed phy regulates genes controlling cytokinesis, the final step of cell division that creates two daughter cells (Inzé, 2007; Buschmann and Muller, 2019). EoD FR represses the expression of genes that regulate the phragmoplast, a plant-specific structure composed mainly of microtubules, which directs new cell wall synthesis (Figure 5E). These include *PHRAGMOPLASTIN-INTERACTING PROTEIN 1 (PHIP1)*; ARM domain containing protein kinase *FUSED (FU)*, involved in male meiosis cytokinesis (Oh et al., 2005); and *AURORA 1 (AUR1)* and *AUR2*, serine/threonine kinases indispensable for eukaryotic cell division that associate during mitosis with plant-specific cytoskeletal structures (preprophase band, phragmoplast, nascent cell plate) and are necessary for cytokinesis (Van Damme et al., 2004; Weimer et al., 2016). We also find effective suppression of several kinesin 12 (KIN12) members (*PAKRP1/KIN12A, KIN12B, POK1/KIN12C*, and *POK2/KIN12D*), which are important for phragmoplast formation and function. For instance, loss of KIN12 reduces phragmoplast stability and expansion, while *kin12a;kin12b* double mutants lack a functional phragmoplast (Lee et al., 2007).

Repression is also observed for several genes encoding microtubule-associated proteins involved in cytokinesis (Supplemental Table S2), including kinesins such as *ATK5*, involved in microtubule spindle morphogenesis (Ambrose and Cyr, 2007); and *BUB3.1* and *BUB3.2*, which associate with MAP65 in the midzone, potentially regulating MAP65 affinity to the microtubules (Buschmann and Muller, 2019). Also affected are microtubule end-binding proteins EB1A, EB1B, and EB1C. These proteins form foci at regions where the minus ends of microtubules are gathered during early cytokinesis (Van Damme et al., 2004; Komaki et al., 2010). ATN, an Arabidopsis protein with high-sequence similarity to the maize microtubule-binding protein TANGLED1, involved in the identification of the division plane during mitosis and cytokinesis (Muller et al., 2006), is downregulated at early- and mid-development.

Our study reveals phy regulates basic cellular processes such as DNA replication, DNA repair, and cytokinesis. It also illustrates that phy exerts strong control on leaf growth through simultaneous regulation of processes that act in concert to execute cell proliferation.

### Phytochrome controls the expression of key leaf development modules

Consistent with the cellular data, we found evidence that EoD FR controlled several leaf developmental modules known to regulate cell division and/or expansion (Figure 5F). *AN3* and other members of the AN3/GRF-SWI/SNF module, including *GRF2, GRF4, GRF6, BRM, ANT*, and *WOX1/STF*, are consistently downregulated by EoD FR treatment (Vercruyssen et al., 2014; Jun et al., 2019; Zhang et al., 2019). AN3/GRF-SWI/SNF has been shown to control the expression of *CYCB1;1* and leaf epidermal and mesophyll cell proliferation and expansion (Kawade et al., 2010; Kawade et al., 2013). Interestingly, *an3-4* mutants exhibit a small, narrow-leaf phenotype that resembles the *phyB* mutant (Horiguchi et al., 2011).

Balancing this, EoD FR enhances the expression of DA1-EOD1 module genes that restrict cell proliferation including *SOD7*, and the *DA1* homologs, *DAR5* and *DAR7* (Li et al., 2008; Zhang et al., 2015). Likewise, HD-Zip II gene *ATHB-2*, previously implicated in shade-induced early exit from cell proliferation, and its homolog *HAT2*, are both upregulated by EoD FR (Carabelli et al., 2018). Thus, phy appears to exert opposing control on positive (AN3/GRF-SWI/SNF), and negative (DA1-EOD1, HD-ZIP II) regulators of cell division, which may explain why phyB is such a potent regulator of leaf growth. Further, increased expression of HD-Zip class I ATHB16 may play a role in limiting cell expansion. Interestingly, transgenic plants expressing its homolog ATHB6 are phenotypically similar, so may also be playing a role in cell expansion (Wang et al., 2003; Henriksson et al., 2005). Of note, *BB* was only upregulated on d20^18^. Overexpression of this factor leads to reduced blade areas and smaller cell size (Disch et al., 2006).

Almost half (48%) of the pavement cells in Arabidopsis leaves are the result of asymmetric divisions of meristemoids (Geisler et al., 2000). It is therefore notable that *PPD2*, a repressor of meristemoid asymmetric division, is upregulated by EoD FR, while *SPCH*, a promoter of meristemoid asymmetric division, plus *FLP* and *FAMA*, which regulate consecutive steps in guard cell development, are downregulated by EoD FR (Figure 5F). Recently, AN3 has also been shown to promote stomatal asymmetric cell division via the transcriptional regulation of COP1 (Meng et al., 2018). PhyB binding to SPA1 is known to disrupt COP1-SPA1 binding and COP1-SPA1 E3 ligase activity (Podolec and Ulm, 2018). As AN3 expression is regulated by EoD FR, this presents a potential alternative pathway via which phyB may alter COP1 signaling and asymmetric stomatal cell division.

Finally, phy also controls the expression of adaxial–abaxial fate and genes including *PHB, AS1, AS2, KAN2*, *ANT, BOP1*, and *BOP2* (Emery et al., 2003; Xu et al., 2003; Nole-Wilson and Krizek, 2006; Ha et al., 2007)*.* In seedlings, *BOP1* and *BOP2* have been reported to have a role in light signaling and have been shown to modulate PIF4 abundance by targeting it for ubiquitin-mediated degradation (Zhang et al., 2017). It might be possible that BOP1 and BOP2 retain a similar regulatory function in the leaf.

Thus, light stable phy appears to control leaf growth and development by regulating the expression of principal leaf development modules genes that serve as master regulators of cell fate, cell proliferation/expansion, meristemoid cell division, and cell polarity.

### Phy action is required to sustain ribosome biogenesis and translation through leaf development

An earlier study demonstrated that phyB is able to regulate translation in the cytosol (Paik et al., 2012). The active (Pfr) form of phyB was shown to interact with PENTA1 (PNT1), which in turn binds to the 5′ untranslated region (5′-UTR) of protochlorophyllide (PORA) mRNA to block its translation. Our bioinformatics analysis expands this concept and reveals phy controls large numbers of genes involved in translation and also the basic cellular translational machinery. For instance, we observe strong repression of aminoacyl-tRNA biosynthesis, elongation factors and almost all of the large and small ribosome subunit genes later in leaf development (Figure 6; Supplemental Figure S9 and Supplemental Table S2). One possible explanation for this striking effect might be that as EoD FR-treated leaves exhibit an early halt to cell division and/or cell expansion, which may reduce the demand for new proteins. Indeed, ribosome abundance and protein synthesis are known to be higher in younger growing leaves, reducing as leaves mature (Ishihara et al., 2015). As ribosomes account for a substantial proportion of cellular protein, the switching down of ribosome gene expression may be an important energy conservation adjustment in EoD FR-exposed leaves that have a shortened growth phase (Ishihara et al., 2017). An alternative and potentially complementary reason for the repression of ribosome formation and translation is EoD FR induction of premature senescence. Recent studies have shown phyB inhibits dark-induced leaf senescence by constraining the levels and activity of PIF4 and PIF5 (Sakuraba et al., 2014; Kim et al., 2020). Consistent with this notion, we observe increased expression of autophagy genes and components of the ethylene (*ERS2* and *EIL*) and ABA (*ABI5* and *EEL*) pathways that have previously been implicated in PIF4/5 senescence induction. Collectively, the data clearly show phy status has a sizeable impact on regulation of the leaf translational apparatus later on in L3 development.

## Conclusions

**Figure 7:**
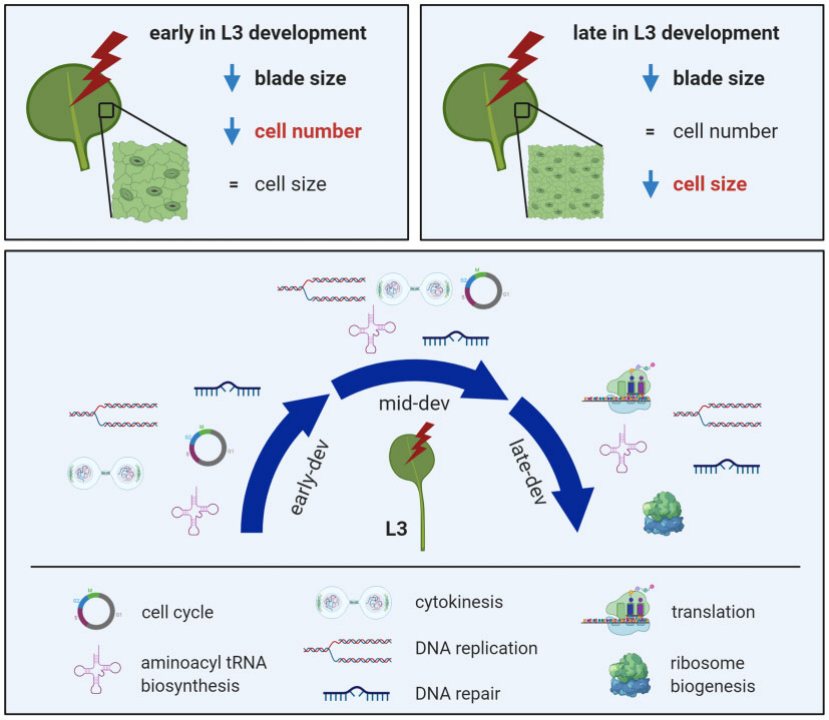
Summary schematic of PhyB mediated control of leaf blade architecture. The SAS blade cellular response is entirely contingent on the timing of FR (red lightning bolt). Early EoD FR exposure limits cell division, while later EoD FR limits cell expansion. Further, phyB action is not confined to hormone signaling, but regulates fundamental aspects of leaf development and physiology: phyB is a master regulator of cell proliferation, exerting simultaneous control on cell cycle, DNA replication, DNA repair, and cytokinesis. Moreover, phyB is a potent regulator of ribosome biogenesis and translation, particularly in late-stage leaf development. L3 = Leaf 3; dev = development.

In summary, our work demonstrates that Arabidopsis leaves exhibit exquisite cellular response plasticity to vegetation shading by employing alternative growth limitation strategies (Figure 7). Phy deactivation by FR-rich vegetation restricts leaf blade growth, either by restraining cell division or cell expansion (depending on when during leaf development shading occurs), mainly through phyB action. Previous blade transcriptomic studies of plants with SAS focused mainly on the role of hormone pathways. Our L3 mRNAseq time series coupled to a stringent bioinformatics analysis pipeline has confirmed those results and further enabled the identification of previously unknown phy signaling paths. This analysis has shown that while expected, well-characterized, light-response genes are upregulated by EoD FR, an overwhelming number of BPs are repressed. Moreover, our study illustrates that phys coordinately regulate cell cycle, DNA replication, DNA repair, and cytokinesis, which are all essential components of cell division. It identified several principal leaf development pathways that are phy regulated and showed phy action has a profound impact on translational machinery. Within the leaf, phys operates as master regulators of development, and of ribosome subunit genes, which may be an energy-conserving measure. A summary schematic can be seen in Figure 7. To facilitate access to and analysis of our mRNAseq data, we have created and online application which can be found at https://aromanowski.shinyapps.io/leafdev-app/.

## Materials and methods

### Plant material and growth conditions

The wild-type Arabidopsis (*A. thaliana*) accession Columbia-0 (Col-0) and the mutant allele *phyB*-9 (Reed et al., 1993) used in this work were obtained from The Nottingham Arabidopsis Stock Centre (NASC). Transgenic *A. thaliana* (Col-0) carrying the pCYCB1;1::D-Box:GUS-GFP reporter (Eloy et al., 2011) construct was kindly provided by Prof Dirk Inzé (PSB, VIB-UGent, The Netherlands).

For all experiments, seeds were stratified in a 0.1% w/v Agar solution, in darkness for 5 d at 4°C. Reagents were purchased from Merck KGaA (Darmstadt, Germany). Seeds were then sown on F2 + S Levington Advance Seed and Modular Compost plus Sand soil mix (ICL Specialty Fertilizers, Suffolk, UK) and grown inside a Percival SE-41L cabinet (CLF Plant Climatics, Wertingen, Germany) under a light: dark (LD) 12-h: 12-h photoperiod, at 100 µmolm^−2^ s^−1^ fluence rate and 21°C of constant temperature. On the 6th d, plants were thinned to a density of 1–2 plants per pot, and then further thinned to 1 plant per pot on the 10th d. Unless otherwise specified, 12 biological replicates/genotypes/conditions were used. Polylux XLR FT8/18W/835 fluorescent tubes (GE) were used as a white light (WL) light source. For EoD FR treatments, we used 7 24V OSLON 150 ILS-OW06-FRED-SD111 FR led strips (Intelligent Led Solutions, Berkshire, UK), to deliver 40 µmol m^−2^ s^−1^ of FR light (730 nm) for 10 min. The spectrum of both light sources can be found in Supplemental Figure S10. Further growth conditions details are provided in the respective figure legends.

### Generation of leaf blade imprints and transparent leaf blades for imaging

To generate leaf blade imprints, leaves (34 d after sowing (D.A.S)) were stuck to a tape with the adaxial epithelial cell layer facing the tape. A single layer of transparent nail varnish (60 Seconds Super Shine 740 Clear, Rimmel, France) was applied over the abaxial epithelial cell layer and left to dry for 30 min. A transparent Sellotape Super Clear Tape (#293616, Sellotape, UK) was stuck to the leaf and slowly peeled off to obtain the full leaf imprint, which was taped to a 76 × 26 mm microscope slide (Menzel-Gläzer, Braunschweig, Germany).

To generate cleared leaf blades, a protocol adapted from (Katagiri et al., 2016) was employed. Briefly, fixation was performed in an eppendorf tube with 1 mL of a mixture of ethanol and acetic acid (6:1) for 4 h. The samples were then washed with 1 mL 70% ethanol for 5 min and then incubated overnight in 400 µL of chloral hydrate solution (8 g: 1 mL: 2 mL chloral hydrate: glycerol: water; Weigel and Glazebrook, 2002) for further clearing. Using chloral hydrate solution as the mounting media, cleared leaf blades were mounted on microscope slides with the adaxial layer facing down.

### Blade area determination

Whole leaf pictures (34 D.A.S) for blade area measurements, including scale bar, were taken using a Nikon G20 camera with automatic focus settings. Blade area was determined using ImageJ (National Institutes of Health).

### Epithelial cell parameter determination

For abaxial epithelial cell parameter determination, leaf imprints and/or cleared blades (34 D.A.S) were mounted and visualized using Eclipse E600 (Nikon) DIC microscope using either a 10X or a 20X objective. Individual abaxial epithelial cell sizes were obtained with ImageJ (NIH). Average leaf cell sizes were obtained by deriving the mean values of 10 adjacent cells from the base, middle, and tip sections of each leaf, or these sections combined. Average total number of cells was obtained by dividing the blade area by the total cell size of each blade, and then averaging the mean total number of cells of each blade. Average cell density was obtained by dividing the total number of cells by the blade area and then averaging the mean cell density of each blade. An S8 stage mic 1 mm/0.01 mm DIV graticule (#02A00404, Pyser-SGI Ltd., Kent, UK) was used for scaling.

### GUS staining

Whole Arabidopsis plants were GUS stained using a protocol adapted from (Weigel and Glazebrook, 2002). Briefly, plants were harvested and incubated in 90% acetone overnight, and, subsequently, washed in wash buffer (50 mM phosphate buffer (pH 7.4), 2 mM K_3_[Fe(CN)_6_], 2 mM K_4_[Fe(CN)_6_], 0.2% v/v Triton X-100) and then incubated in 5‐bromo‐4‐chloro‐3‐indolyl‐β‐glucuronide (X‐Gluc) buffer (wash buffer supplemented with 2 mM X‐Gluc in N,N-dimethylformamide) at room temperature for 48 h. Samples were washed and cleared for 30-min intervals in an increasing ethanol series (i.e., 35%, 50%, 70% ethanol) and further cleared overnight in chloral hydrate solution. Samples were then photographed under a Leica MZ 16 F dissecting microscope.

### qPCR gene expression analysis

For RT-qPCR experiments, whole 13 D.A.S. Col-0 seedlings (150 per replicate) or 16 and 20 D.A.S Col-0 third leaves (90 and 40 per replicates, respectively) were first collected at ZT22 and submerged in RNA Later, then using a Leica MZ 16 F dissecting microscope, L3 primordia or blades were dissected out with a scalpel (Supplemental Figure S11) and placed again in RNA Later solution. Total RNA was extracted using the RNeasy Plant Mini Kit (Qiagen) with on-column DNase digestion. All samples were processed on the same day. cDNA synthesis was performed using the qScript cDNA SuperMix (Quanta Biosciences) as described by the manufacturer. The RT-qPCR was set up as a 10 μL reaction using Lightcycler 480 SYBR Green Master Mix (Roche) in a 384-well plate, performed with a Lightcycler 480 system (Roche). Results were analyzed using the Light Cycler 480 software. The primers used in this study are listed in Supplemental Table S7.

### cDNA library preparation and high-throughput sequencing

Total RNA was extracted from Col-0 plants as described above. Samples were then sent to Edinburgh Genomics for QC check and sequencing. Sample quality was checked using Qubit with the broad range RNA kit (Thermo Fisher Scientific) and Tapestation 4200 with the RNA Screentape for eukaryotic RNA analysis (Agilent). Libraries were prepared using the TruSeq Stranded mRNA kit (Illumina) and then validated. Samples were pooled to create 14 multiplexed DNA libraries, which were paired-end (PE) sequenced on an Illumina HiSeq 4000 platform (Machine name K00166, Run number 346, flowcell AHT2HKBBXX, lanes 5 and 6). On average, 23.9 million 150 nt PE reads were obtained for each sample (Supplemental Table S8).

### Processing of RNA sequencing reads

Raw sequence reads were trimmed with cutadapt 1.9.1 (Martin, 2011) with default parameters and—a set to ‘AGATCGGAAGAGC’, to eliminate adapter contamination from the PE reads. Trimmed reads were aligned against the *A. thaliana* genome (TAIR10) with TopHat v2.1.1 (Kim et al., 2013) with default parameters, except in the case of the maximum intron length parameter, which was set at 5,000 (Supplemental Table S8). Count tables for the different feature levels were obtained from bam files using custom R scripts and considering the AtRTD2 transcriptome (Zhang et al., 2017). Briefly, for this purpose, we used the ‘ASpli::readCounts()’ function of ASpli package version 1.8.1 (Mancini et al., 2021), which uses the GenomicFeatures Bioconductor package (Lawrence et al., 2013). Count tables at the gene level presented a good correlation overall between replicates and samples (Supplemental Figure S12). Raw sequences (fastq files) used in this paper have been deposited in the ArrayExpress (Kolesnikov et al., 2015) database at EMBL-EBI (www.ebi.ac.uk/arrayexpress) under accession number E-MTAB-9445.

### DGE analysis

DGE analysis was conducted using custom R scripts for 18,934 genes whose expression was above a minimum threshold level (read density >0.05 and at least 10 counts-per-million) in at least one experimental condition. Read density was computed as the number of reads in each gene divided by its effective width. The term ‘effective width’ corresponds to the sum of the length of all the exons of a given gene. DGE was estimated using the edgeR package version 3.22.3 (Robinson et al., 2010; Lun et al., 2016), and resulting *P* values were adjusted using a false discovery rate (FDR) criterion. Genes with FDR values lower than 0.1 or 0.05 and an absolute log_2_ fold change >0.58 were considered differentially expressed. Heatmaps were generated using R or GraphPad Prism 7 (GraphPad Software).

### GO and KEGG metabolic pathway analysis

Gene set enrichment analysis was performed using a combination of custom written R scripts and the GOstats Bioconductor package version 3.9 (Falcon and Gentleman, 2007). GO enrichment analysis was performed using the 18,934 expressed genes as the universe gene set. GO terms with *P* < 0.05 and FDR < 0.1 were summarized to remove redundant GO terms using REVIGO (Supek et al., 2011) with default values, small allowed similarity (0.5) and the ‘*Arabidopsis thaliana*’ database for GO term sizes. Bubble plots were generated using R. KEGG pathway enrichment was analyzed using R and the clusterProfiler package (Yu et al., 2012) version 3.16.1 of Bioconductor. All the pathways of *A. thaliana* were derived from the KEGG Pathway Database (http://www.kegg.jp; Kanehisa and Goto, 2000; Kanehisa et al., 2012). KEGG enrichment analysis was performed using the 18,934 expressed genes as the gene universe set and those with *P* < 0.05 and FDR <0.1 were furthered considered. Individual KEGG pathways were visualized utilizing the Pathview package (Luo and Brouwer, 2013) version 1.28 of Bioconductor.

### Statistical analysis

Statistical difference of two populations was tested by two-tailed, unpaired Student’s *t* test. To compare three or more populations, one-way analysis of variance (ANOVA) followed by Dunnett’s test (comparison against a control) or Tukey’s test (comparison among all groups) was performed. When Tukey’s test was employed, letters were used to indicate which treatment groups were significantly different. All analyses were done using GraphPad Prism 7 (GraphPad Software) or Minitab 18 (Minitab Ltd.), unless otherwise indicated.

### Accession numbers and data availability

Raw sequences (fastq files) used in this paper have been deposited in the ArrayExpress (Kolesnikov et al., 2015) database at EMBL-EBI (www.ebi.ac.uk/arrayexpress) under accession number E-MTAB-9445.

All custom R scripts are available in https://github.com/aromanowski/leaf3_dev and https://github.com/aromanowski/LeafDev-app. Alternatively, they are available upon request to the corresponding author.

## Supplemental data

The following materials are available in the online version of this article.

**Supplemental Figure S1.** Techniques used to visualize epithelial cells and abaxial epithelial cell size and density by spatial location.

**Supplemental Figure S2.** Effect of EoD FR treatment on L3 blade parameters and expression of the pCYCB1;1–GUS reporter gene at different developmental stages.

**Supplemental Figure S3.** qPCR validation of classic shade response genes.

**Supplemental Figure S4.** Smear plots and Venn diagram analysis of gene expression.

**Supplemental Figure S5.** Effect of FDR on DGE analysis

**Supplemental Figure S6.** EoD FR modulation of plant transcription factors and hormone signal transduction pathways.

**Supplemental Figure S7.** DNA repair pathways affected by EoD FR.

**Supplemental Figure S8.** Leaf developmental genes affected by EoD FR.

**Supplemental Figure S9.** Translation processes affected by EoD FR treatment.

**Supplemental Figure S10.** Spectral data information.

**Supplemental Figure S11.** Diagram of leaf tissue dissection for RNA extraction.

**Supplemental Figure S12.** Correlation between RNA-seq samples.

**Supplemental Table S1.** Normalized logCPM gene expression values.

**Supplemental Table S2.** Differentially expressed genes.

**Supplemental Table S3.** GO terms analysis.

**Supplemental Table S4.** REVIGO summarization of GO terms.

**Supplemental Table S5.** Transcription factors affected by EoD FR.

**Supplemental Table S6.** KEGG pathways analysis.

**Supplemental Table S7.** Primers used in this work.

**Supplemental Table S8.** Mapping statistics.

## Supplementary Material

kiab112_Supplementary_Data

## Notes

A.R. contributed to the following: conceptualization, methodology, software, formal analysis, investigation, data curation, writing—original draft, writing—review and editing, visualization, and supervision. James J. Furniss contributed to the following: conceptualization, methodology, validation, formal analysis, investigation. Ejaz Husain contributed to the following: validation and investigation. Karen J. Halliday contributed to the following: conceptualization, resources, writing—original draft, writing—review and editing, supervision, project administration, and funding acquisition.

The author responsible for distribution of materials integral to the findings presented in this article in accordance with the policy described in the Instructions for Authors (https://academic.oup.com/plphys/pages/general-instructions) are: Karen J. Halliday (Karen.Halliday@ed.ac.uk) and Andrés Romanowski (Andrew.Romanowski@ed.ac.uk).
