# Supplementary material for: Phytochrome regulates cellular response plasticity and the basic molecular machinery of leaf development
Source: Plant Physiol. 2021 Mar 9;186(2):1220–39. doi: 10.1093/plphys/kiab112 (PMC8195529; doi:10.1093/plphys/kiab112)
Supplement: kiab112_Supplementary_Data [file kiab112_supplementary_data.zip › Supplemental_data-AR_final.pdf]

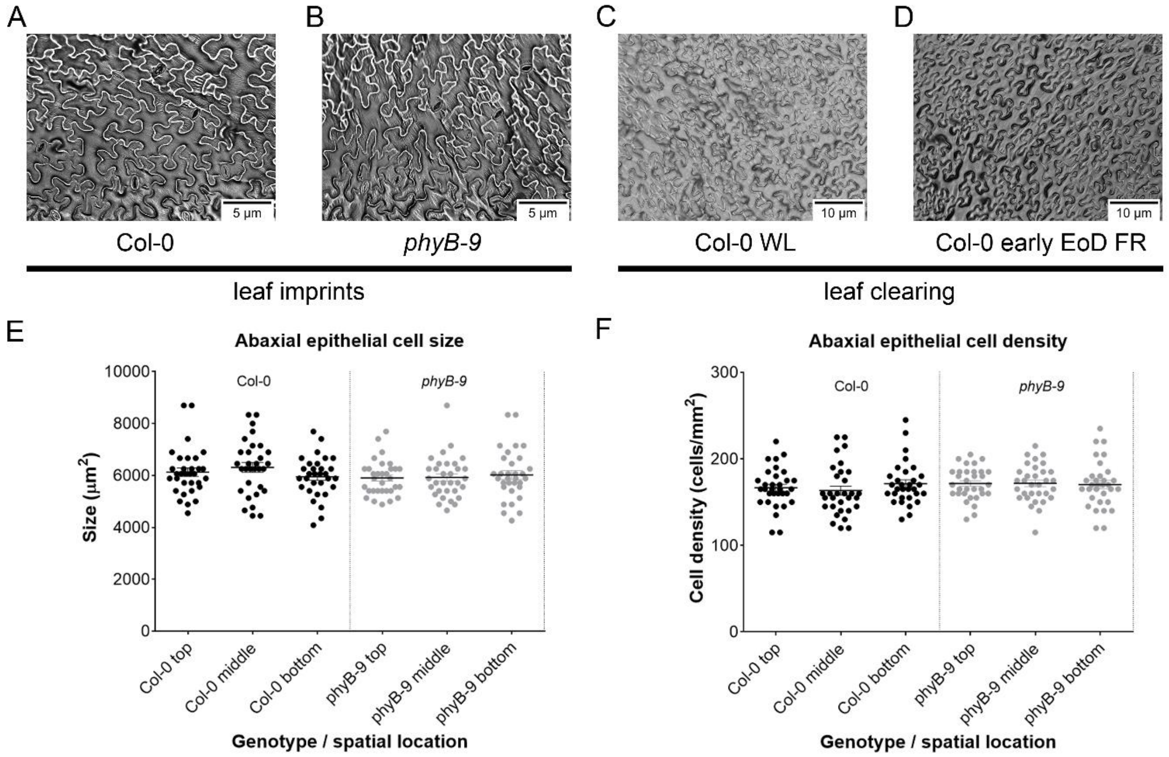

Supplemental Figure S1. Techniques used to visualize epithelial cells and abaxial epithelial cell size and density by spatial location. (A-B) DIC microscopy image of leaf imprints of Col-0 (A) and phyB-9 (B) abaxial side of L3. The black bar is 5 µm. **(C-D)** DIC microscopy image of leaf clearing of the abaxial side of L3 of Col-0 plants under WL (C) or EoD FR (D) conditions. The black bar is 10 µm. **(E-F)** The plot shows the distribution of abaxial epithelial cell sizes (E) or cell density (F) in L3 of Col-0 (black) and phyB-9 (grey) at the top (tip), middle, or bottom of the leaf blade. Means were compared by one-way ANOVA followed by Tukey's multiple comparisons test (n = 32 cells per spatial location, n.s.; GraphPad Prism). Error bars indicate SEM. WL = white light; EoD = end of day; FR = far-red.

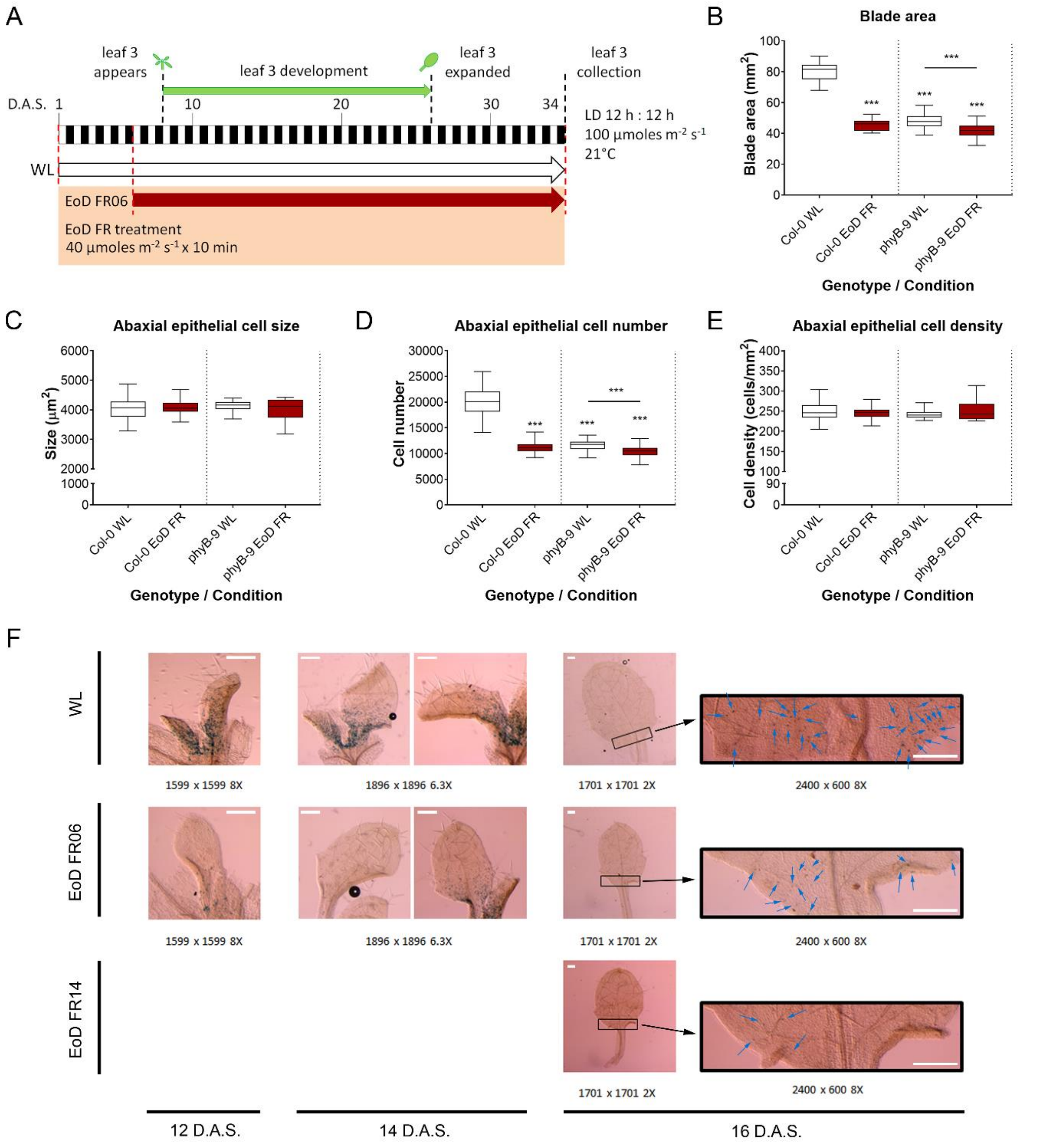

Supplemental Figure S2. Effect of EoD FR treatment on L3 blade parameters and expression of the pCYCB1;1–GUS reporter gene at different developmental stages. (A) Schematic representation of the environmental treatment regime. White rectangles indicate the 12 h of day period. Black rectangles indicate the 12 h of dark period. The green arrow indicates the period of L3 development, which is enclosed between two black dashed lines. The plant drawn on top of day 8 indicates L3 emergence. The leaf drawn on top of day 26 indicates that L3 is fully expanded. The red dashed lines indicate the day at which a specific treatment was started and coincides with a specific-colored arrow (WL in white and EoD FR 06 in dark red). The red dashed line at the end of day 34 marks the end of each treatment. The black dashed line on day 34 indicates tissue collection. **(B-E)** EoD FR treated Col-0 L3s are similar to phyB-9, as can be seen by EoD FR effects on blade area (n = 20 leaves per condition; *** = $p < 0.001$ vs Col-0 WL and phyB-9 EoD FR vs phyB-9 WL) (B); abaxial epithelial cell size (n = 305 cells per condition; n.s.) (C); abaxial epithelial cell number (n = 20 leaves per condition; *** = $p < 0.001$ vs Col-0 WL and phyB-9 EoD FR vs phyB-9 WL) (D); and abaxial epithelial cell density (n = 20 leaves per condition; n.s.) (E). Means were compared by one-way ANOVA followed by Tukey's multiple comparisons test (GraphPad Prism). In all box plots: center line, mean; box limits, 25th to 75th percentiles; whiskers, min to max; points, outliers. Error bars indicate SEM. **(F)** Microscopy images of GUS stained L3 at different moments of development (12, 14, and 16 D. A. S) grown under the following

environmental conditions: white light (WL), EoD FR since day 6 (EoD FR06), or EoD FR since day 14 (EoD FR14). The numbers below each image indicate size in pixels and magnification. The panel on the right corresponds to a zoom in to the indicated area of the leaf to allow for better visualization of stained cells (marked by blue arrows). White scale bars represent 0.2 mm. L3 = Leaf 3; LD = Light : Dark; D.A.S. = Days After Sowing; WL = white light; EoD = end of day; FR = far-red; EoD FR06 = EoD FR since day 6; EoD FR14 = EoD FR since day 14.

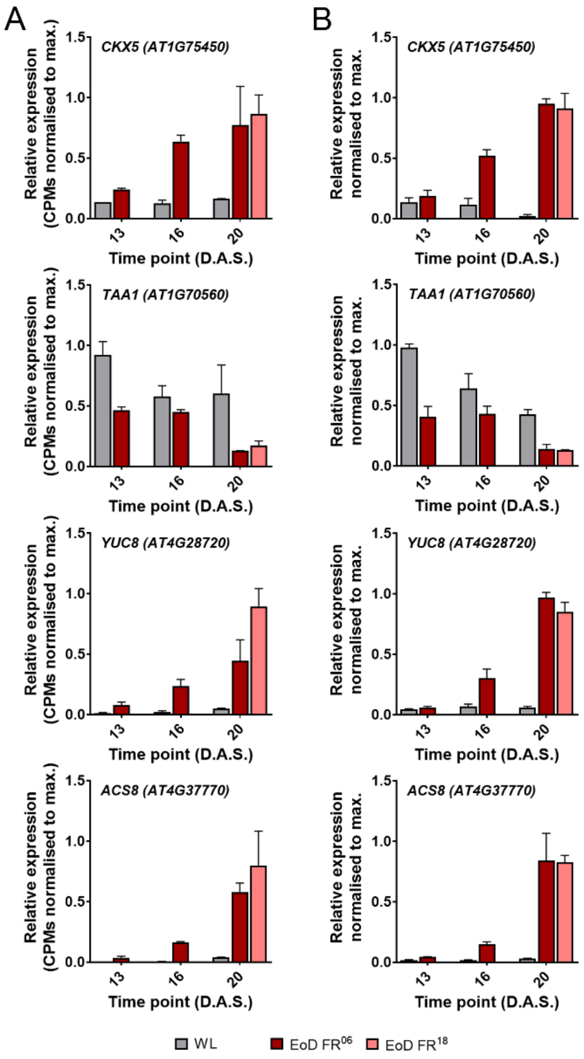

Supplemental Figure S3. qPCR validation of classic shade response genes. (A) Gene expression of known shade responders shown as normalized counts. **(B)** qPCR validation of known shade responders (n = 2 biological replicates with 3 technical replicates per time point per condition). Error bars represent S.E.M. LD = Light : Dark; D.A.S. = Days After Sowing; WL = white light; EoD = end of day; FR = far-red; EoD FR^{06} = EoD FR since day 6; EoD FR^{18} = EoD FR since day 18.

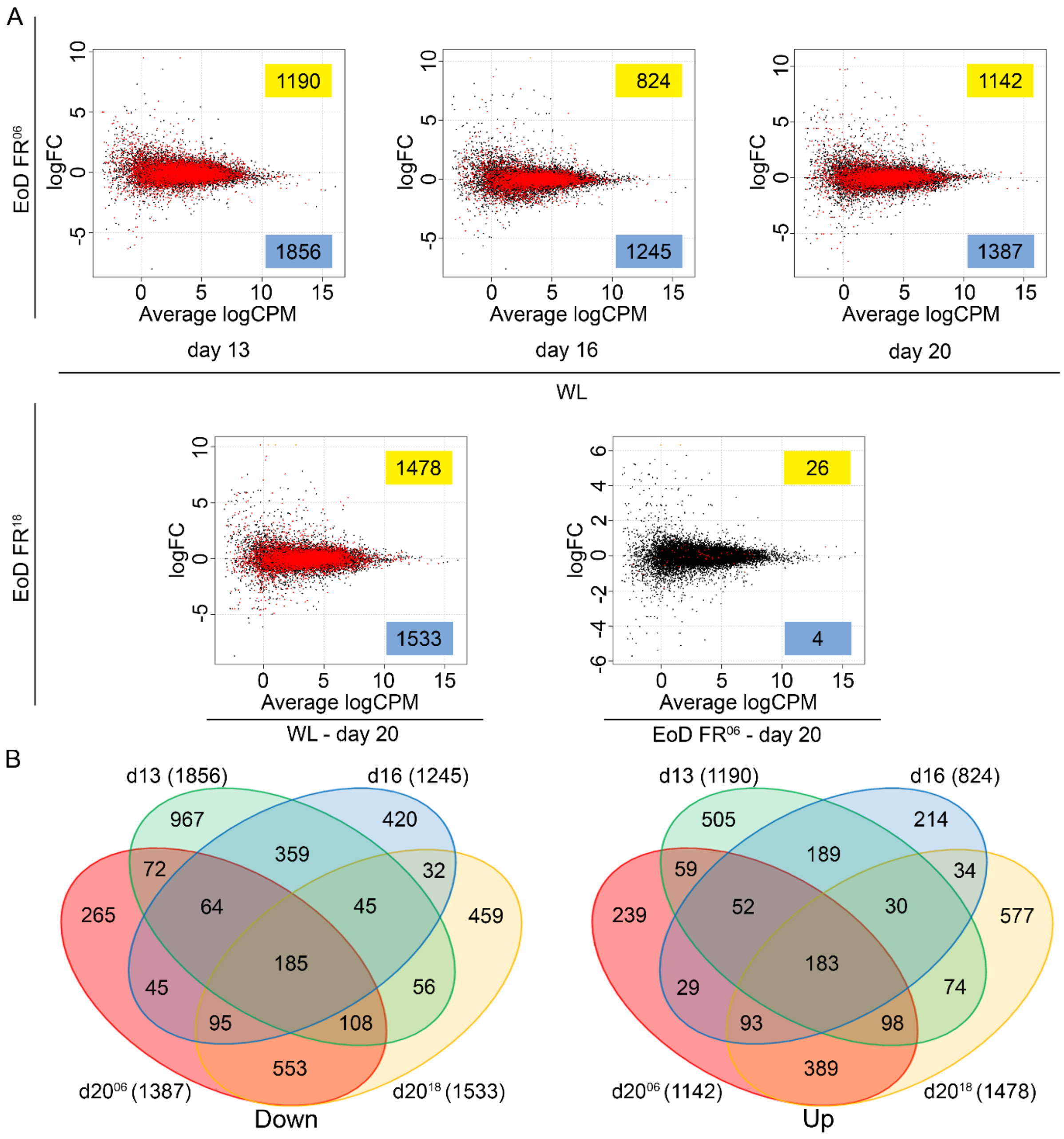

Supplemental Figure S4. Smear plots and Venn diagram analysis of gene expression. (A) Smear plots of the differential gene expression analysis of (top) EoD FR^{06} treated samples compared to WL samples on day 13, day 16, and day 20 (from left to right), and (bottom) EoD FR^{18} treated samples on day 20 compared to WL (left) and EoD FR^{06} (right). Differentially expressed genes are shown in red and non-differentially expressed genes are shown in black. The number of up-regulated and down-regulated genes are shown within yellow and blue boxes, respectively, for each graph. **(B)** Venn diagram of down-regulated (left) and up-regulated (right) differentially expressed genes, showing the overlap between the different time points as compared to their respective samples in WL (control) conditions. D.A.S. = Days After Sowing; WL = white light; EoD = end of day; FR = far-red; EoD FR^{06} = EoD FR since day 6; EoD FR^{18} = EoD FR since day 18. In the Venn diagrams, d13 = day 13 under EoD FR since day 6 treatment; d16 = day 16 under EoD FR since day 6 treatment; $d20^{06}$ = day 20 under EoD FR since day 6 treatment; $d20^{18}$ = day 20 under EoD FR since day 18 treatment.

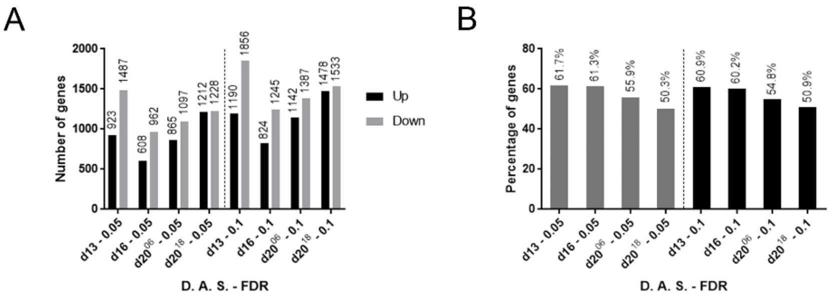

C

BP	all	up	down	down/total
d13 - 0.05	655	30	625	95.4%
d16 - 0.05	497	66	431	86.7%
$d20^{06}$ - 0.05	355	88	267	75.2%
$d20^{18}$ - 0.05	342	94	248	72.5%

MF	all	up	down	down/total
d13 - 0.05	77	0	77	100.0%
d16 - 0.05	100	14	86	86.0%
$d20^{06}$ - 0.05	95	15	80	84.2%
$d20^{18}$ - 0.05	42	14	28	66.7%

CC	all	up	down	down/total
d13 - 0.05	77	0	77	100.0%
d16 - 0.05	52	0	52	100.0%
$d20^{06}$ - 0.05	78	2	76	97.4%
$d20^{18}$ - 0.05	75	4	71	94.7%

D

BP	all	up	down	down/total
d13 - 0.1	605	29	576	95.2%
d16 - 0.1	471	32	439	93.2%
$d20^{06}$ - 0.1	384	76	308	80.2%
$d20^{18}$ - 0.1	394	83	311	78.9%

MF	all	up	down	down/total
d13 - 0.1	52	0	52	100.0%
d16 - 0.1	96	15	81	84.4%
$d20^{06}$ - 0.1	65	2	63	96.9%
$d20^{18}$ - 0.1	33	12	21	63.6%

CC	all	up	down	down/total
d13 - 0.1	70	0	70	100.0%
d16 - 0.1	55	3	52	94.5%
$d20^{06}$ - 0.1	77	3	74	96.1%
$d20^{18}$ - 0.1	70	4	66	94.3%

Supplemental Figure S5. Effect of FDR on differential gene expression analysis. A) Number of mis regulated genes (differentially expressed genes) per time point (dark bars indicate up regulated genes, grey bars indicate down regulated genes) using an FDR < 0.05 (left of the dashed line) or an FDR < 0.1 (right of the dashed line). B) Time point analysis of the percentage of downregulated genes as compared to the total number of mis regulated genes using an FDR < 0.05 (left of the dashed line, grey bars) or an FDR < 0.1 (right of the dashed line, black bars). C-D) Comparison of Gene Ontology (GO) Terms by timepoint, affected by down regulation or up regulation ($p < 0.05$ and $q < 0.1$; BP = Biological Process, MF = Molecular Function, CC = Cellular Component) of differentially expressed genes with an FDR < 0.05 (C) or an FDR < 0.1 (D). d13 = day 13 under EoD FR since day 6 treatment; d16 = day 16 under EoD FR since day 6 treatment; $d20^{06}$ = day 20 under EoD FR since day 6 treatment; $d20^{18}$ = day 20 under EoD FR since day 18 treatment.

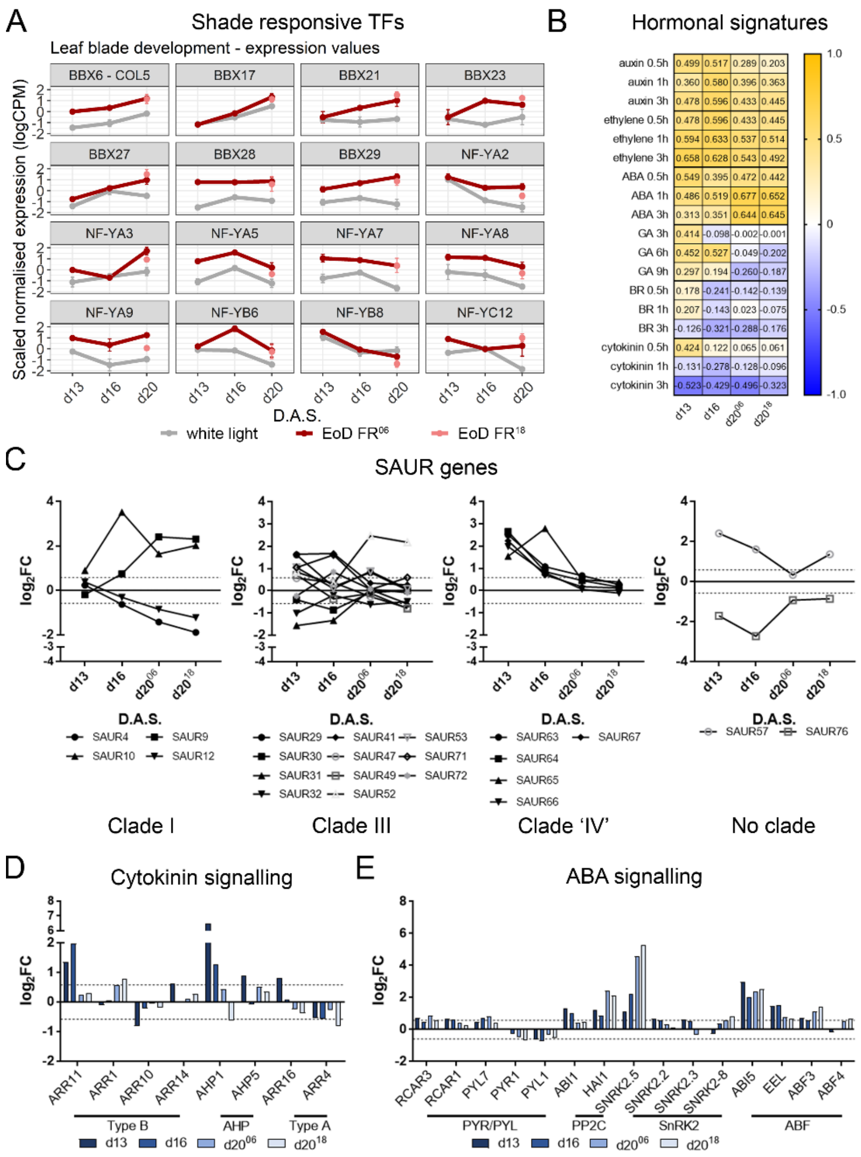

Supplemental Figure S6. EoD FR modulation of plant transcription factors and hormone signal transduction pathways. (A) Gene plots of TF affected by EoD FR under white light (grey), EoD FR^{06} treatment (dark red), and EoD FR^{18} treatment (pink) across all time points (error bars indicate SEM). **(B)** Hormonometer analysis of the L3 transcriptome (Volodarsky et al., 2009). Numbers indicate the correlation ranks. **(C)** Line plots of log_2FC differential gene expression values of (from left to right) Clade I, Clade III, Clade 'IV' and SAURs not assigned to any clade. **(D-E)** Bar plots of cytokinin (D) and abscisic acid (ABA) (E) signaling genes affected by EoD FR. The dashed lines (C-E) indicate the $|log_2FC| = 0.58$ threshold. D.A.S. = Days After Sowing; EoD = end of day; FR = far-red; EoD FR06 = EoD FR since day 6; EoD FR18 = EoD FR since day 18; d13 = day 13 under EoD FR since day 6 treatment; d16 = day 16 under EoD FR since day 6 treatment; $d20^{06}$ = day 20 under EoD FR since day 6 treatment; $d20^{18}$ = day 20 under EoD FR since day 18 treatment.

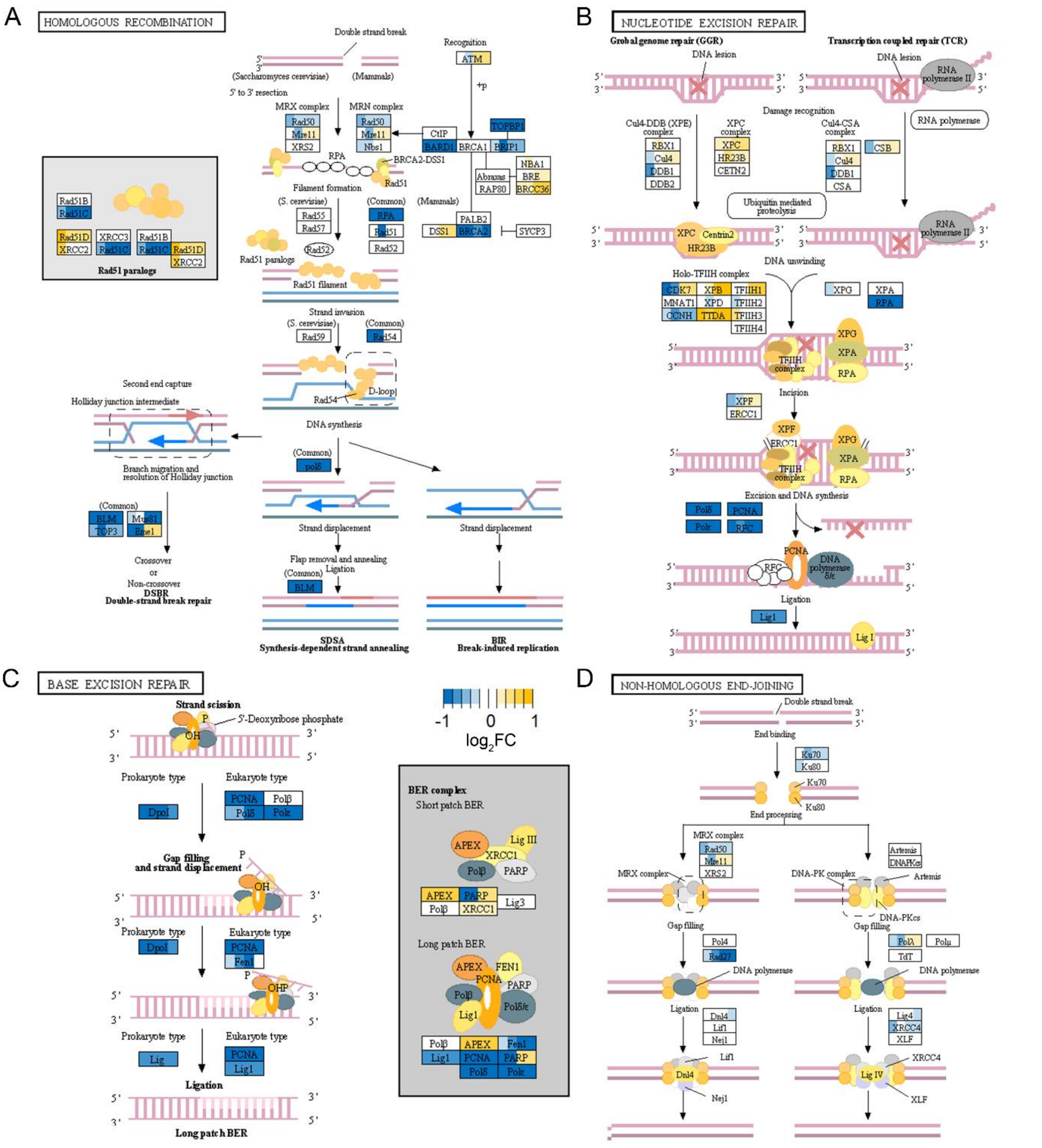

Supplemental Figure S7. DNA repair pathways affected by EoD FR. (A-D) Modified schematic of the homologous recombination (ath03440) (A), nucleotide excision repair (ath03420) (B), base excision repair (ath03410) (C), and non-homologous end-joining (ath03450) (D) KEGG *Arabidopsis thaliana* metabolic pathways. Original KEGG Graph data (Kanehisa and Goto, 2000) were rendered with the Pathview R package (Luo and Brouwer, 2013). Each rectangle is divided into 4 color regions reflecting the scaled log_2FC value of each time point. The regions are arranged from left to right representing d13, d16, $d20^{06}$, and $d20^{18}$, respectively.

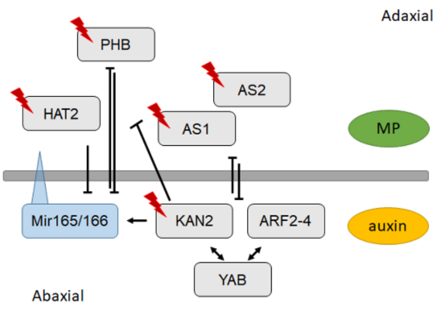

Supplemental Figure S8. Leaf developmental genes affected by EoD FR. Simplified schematic of abaxial-adaxial axis patterning determining factors. The grey line indicates the boundary between the adaxial and abaxial sides. The red lightning bolt icons indicate that the gene coding for this factor is affected by EoD FR.

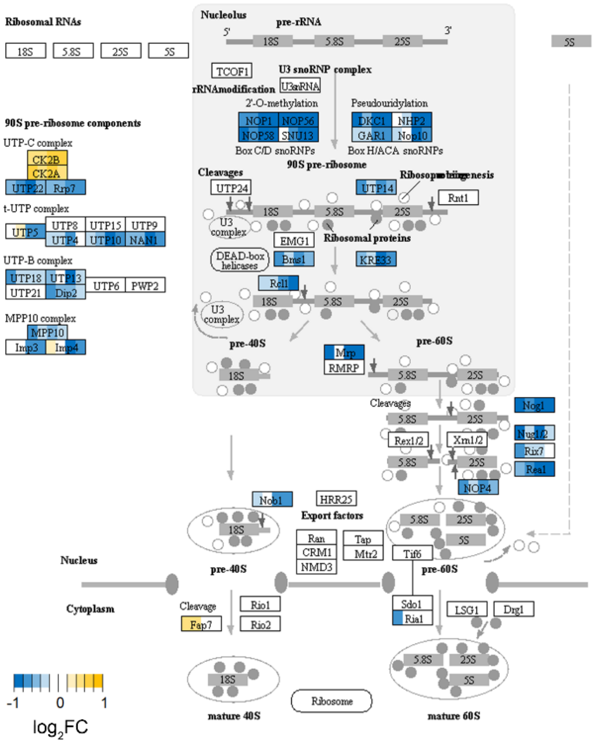

Supplemental Figure S9. Translation processes affected by EoD FR treatment. Modified schematic of the ribosome biogenesis (ath03008) KEGG *Arabidopsis thaliana* metabolic pathways. Original KEGG Graph data (Kanehisa and Goto, 2000) were rendered with the Pathview R package (Luo and Brouwer, 2013). Each rectangle is divided into 4 color regions reflecting the scaled log_2FC value of each time point. The regions are arranged from left to right representing d13, d16, $d20^{06}$, and $d20^{18}$, respectively.

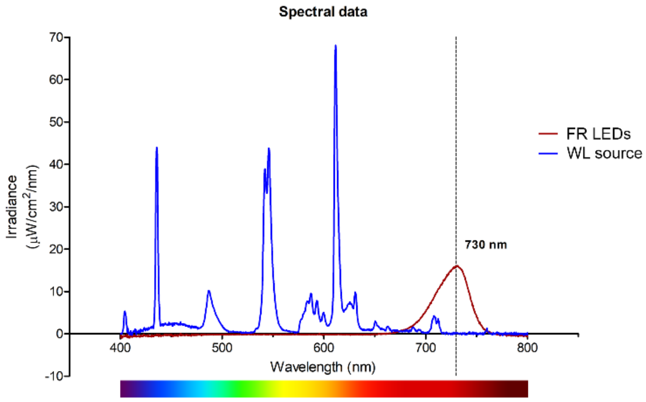

Supplemental Figure S10. Spectral data information. Plot of irradiance for the white light (WL) source (blue) and far-red (FR) LEDs (dark red) used in this work. The dashed line marks the peak of FR emission (730 nm).

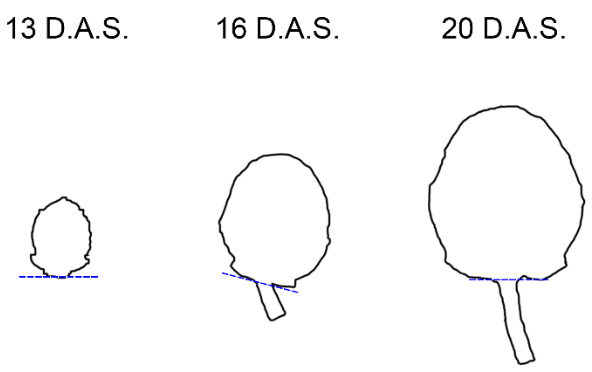

Supplemental Figure S11. Diagram of leaf tissue dissection for RNA. Schematic diagram depicting the boundaries (dotted blue line) of the dissection of leaf blade tissue samples used in the qPCR and mRNA-seq assays. D.A.S. = Days After Sowing.

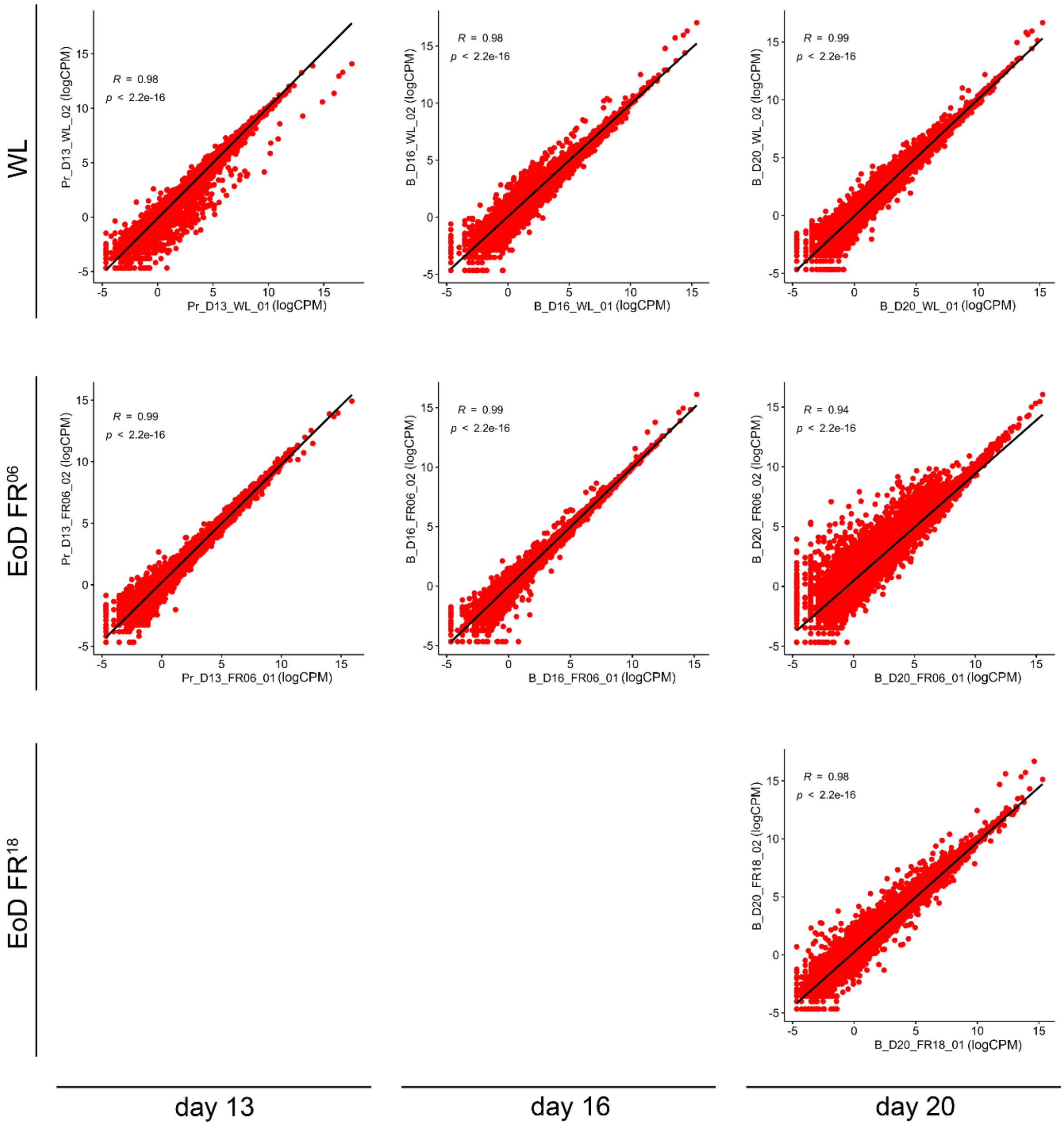

Supplemental Figure S12. Correlation between RNA-seq samples. Replicate correlation analysis and linear regression (dark line) of (from top left to bottom right): day 13 WL, day 16 WL, day 20 WL, day 13 EoD FR^{06}, day 16 EoD FR^{06}, day 20 EoD FR^{06}, and day 20 EoD FR^{18} samples. WL = white light; EoD = end of day; FR = far-red; EoD FR06 = EoD FR since day 6; EoD FR18 = EoD FR since day 18.
